# Varied-shaped gold nanoparticles with nanogram killing efficiency as potential antimicrobial surface coatings for the medical devices

**DOI:** 10.1038/s41598-021-91847-3

**Published:** 2021-06-15

**Authors:** Ewelina Piktel, Łukasz Suprewicz, Joanna Depciuch, Sylwia Chmielewska, Karol Skłodowski, Tamara Daniluk, Grzegorz Król, Paulina Kołat-Brodecka, Piotr Bijak, Anna Pajor-Świerzy, Krzysztof Fiedoruk, Magdalena Parlinska-Wojtan, Robert Bucki

**Affiliations:** 1 Department of Medical Microbiology and Nanobiomedical Engineering, Medical University of Bialystok, Mickiewicza 2c, 15-222 Białystok, Poland; 2 Institute of Nuclear Physics, Polish Academy of Sciences, 31-342 Krakow, Poland; 3 Department of Microbiology and Immunology, Institute of Medical Science, Collegium Medicum, Jan Kochanowski University in Kielce, Aleja IX Wieków Kielc 19A, 25-317 Kielce, Poland; 4 University Clinical Hospital of the Military Medical Academy-Central Veterans Hospital, Żeromskiego 113, 90-549 Łódź, Poland; 5 Jerzy Haber Institute of Catalysis and Surface Chemistry Polish Academy of Sciences, Niezapominajek 8, 30239 Krakow, Poland

**Keywords:** Antimicrobials, Applied microbiology, Microbiology

## Abstract

Medical device-associated infections are a serious medical threat, particularly for patients with impaired mobility and/or advanced age. Despite a variety of antimicrobial coatings for medical devices being explored to date, only a limited number have been introduced for clinical use. Research into new bactericidal agents with the ability to eradicate pathogens, limit biofilm formation, and exhibit satisfactory biocompatibility, is therefore necessary and urgent. In this study, a series of varied-morphology gold nanoparticles in shapes of rods, peanuts, stars and spherical-like, porous ones with potent antibacterial activity were synthesized and thoroughly tested against spectrum of *Candida albicans, Pseudomonas aeruginosa, Staphylococcus aureus* clinical strains, as well as spectrum of uropathogenic *Escherichia coli* isolates. The optimization of gold nanoparticles synthesis allowed to develop nanomaterials, which are proved to be significantly more potent against tested microbes compared with the gold nanoformulations reported to date. Notably, their antimicrobial spectrum includes strains with different drug resistance mechanisms. Facile and cost-efficient synthesis of gold nanoparticles, remarkable bactericidal efficiency at nanogram doses, and low toxicity, underline their potential for development as a new coatings, as indicated by the example of urological catheters. The presented research fills a gap in microbial studies of non-spherical gold nanoparticles for the development of antimicrobial coatings targeting multidrug-resistant pathogens responsible for device-associated nosocomial infections.

## Introduction

Medical device-associated infections are common nosocomial infections, particularly in critically ill patients. The prolonged use of implantable medical devices and urinary catheters is recognized as a primary factor in the increased risk infections and bacteraemia, which both considerably enhance mortality, increase medical costs, and extend hospitalization, particularly among elderly patients. Moreover, drug resistance can develop as a result of prolonged exposure to antibiotics during hospital stays and biofilm formation on medical devices^1^. Device-associated risk is primarily determined by the nature of the substances used for biomaterials fabrication, which allow for microorganism colonization and the formation of biofilms^2^. For this reason, efforts have been made to improve the antibacterial and antifungal features of medical devices by coating them with substances that exhibit bactericidal and anti-biofilm properties^3^. To date, a number of antimicrobial agents with the ability to prevent device-associated infections have been proposed as biomaterials coatings. The most conventional approach is the use of antibiotics or antibiotic-releasing polymers^4,5^, nevertheless, it is ineffective against drug-resistant pathogens and promote drug resistance itself. For this reason, alternative methods are constantly developed and thoroughly tested. A great part of these applications involve the coating of medical devices surfaces by metallic nanoparticles, such as zinc oxide (ZnO NPs)^6^, silver (AgNPs)^7^, copper (CuNPs)^8^ or titanium (TiO_2_ NPs)^9^ and mechanism of protective effects of those nanomaterials includes mostly the disruption of microbial membranes and preventing microbial proliferation of the surface of device or implant. Among them, silver nanoparticles (AgNPs) are the most recognized particularly due to the killing efficiency against wild spectrum of bacterial pathogens, including *Escherichia coli* or *Staphylococcus aureus*^10^ and low cytotoxicity^11^ but also due to promoting the formation of carbonated hydroxyapatite, which is favorable for fabrication of orthopaedic devices^12^. Bacterial-selective, stable and easy to produce zinc oxide nanoparticles were presented as alternative to AgNPs^6^, however, they did not achieved such importance in clinical settings as the silver ones. Some hopes were also related with the use of copper nanoparticles, which were recognized as fungal-targeting^13^, however, some adjustments allowing to control the release of copper, and thus limit human toxicity, are required. In one of the most interesting research, Slamborova et al. combined silver, copper and titanium dioxide nanoparticles to establish long-term, broad-spectrum both antifungal and antibacterial coverage while maintaining the appropriate mechanical properties of the coating itself^14^. Other hybrid coatings were also developed from combining silver, zinc oxide and titanium dioxide nanoparticles^15^. However, despite the range of developed solutions for coating of medical devices, a rate of implants-associated infections is still alarmingly high. This motivated research into novel antimicrobials that exhibit antibacterial and antibiofilm properties and can be effectively used as antimicrobial coatings for medical devices.

Owing to their unique physicochemical features and low cytotoxicity, gold nanoparticles (AuNPs)^16^ have been widely used in biological and biotechnological applications as biocidal agents^17^, drug delivery systems^18^, photosensitizers^19^, and molecular diagnostic tools^20^. The plasma membrane and bacterial DNA are the major targets of AuNPs^21,22^, however, other molecules such as components of apoptosis-like pathways have also been reported as targets^23,24^. The size and shape of gold nanoparticles strongly determine the nature of their interaction with bacterial cells and subsequent internalization into the cytoplasm of pathogens^22^, which allows the bactericidal efficiency of AuNPs to be modified by developing structures with controlled shape and size. In this study, we assess the antimicrobial efficiency of non-spherical gold nanoparticles in shape of rods (AuR NPs), peanuts (AuP NPs) and stars (AuS NPs), as well as porous spherical-like nanoparticles [AuSph (70C) NPs] prepared by modified CTAB (cetrimonium bromide)-assisted method. Notably, the optimization of synthesis method of the non-spherical gold nanoparticles determined their killing efficiency and allowed to obtain remarkably potent antimicrobial compounds, in which we believe lies the novelty of this research. In contrast to previous reports where microgram concentrations (µg mL^−1^) of gold nanoparticles were required to exert a satisfactory bactericidal effect^22,25^, the nanoparticles developed in this study demonstrated highly efficient killing of fungal isolates, as well as Gram-positive and Gram-negative bacteria isolates at nanogram concentrations (ng mL^−1^). More detailed analyses revealed a great potential of developed nanoparticles as bactericidal agents against uropathogenic *E. coli*, which followed by high antibacterial and anti-biofilm activity, both in the presence of urine and variable pH, highlights their potential as urinary catheter coatings. This work therefore provides a promising starting point for further investigation into the biocidal activity of the nanoparticles against other pathogenic microorganisms, and their potential as coating of other medical devices.

## Results

### Synthesis and physicochemical analysis of rod- and peanut-shaped gold nanoparticles

**Figure 1:**
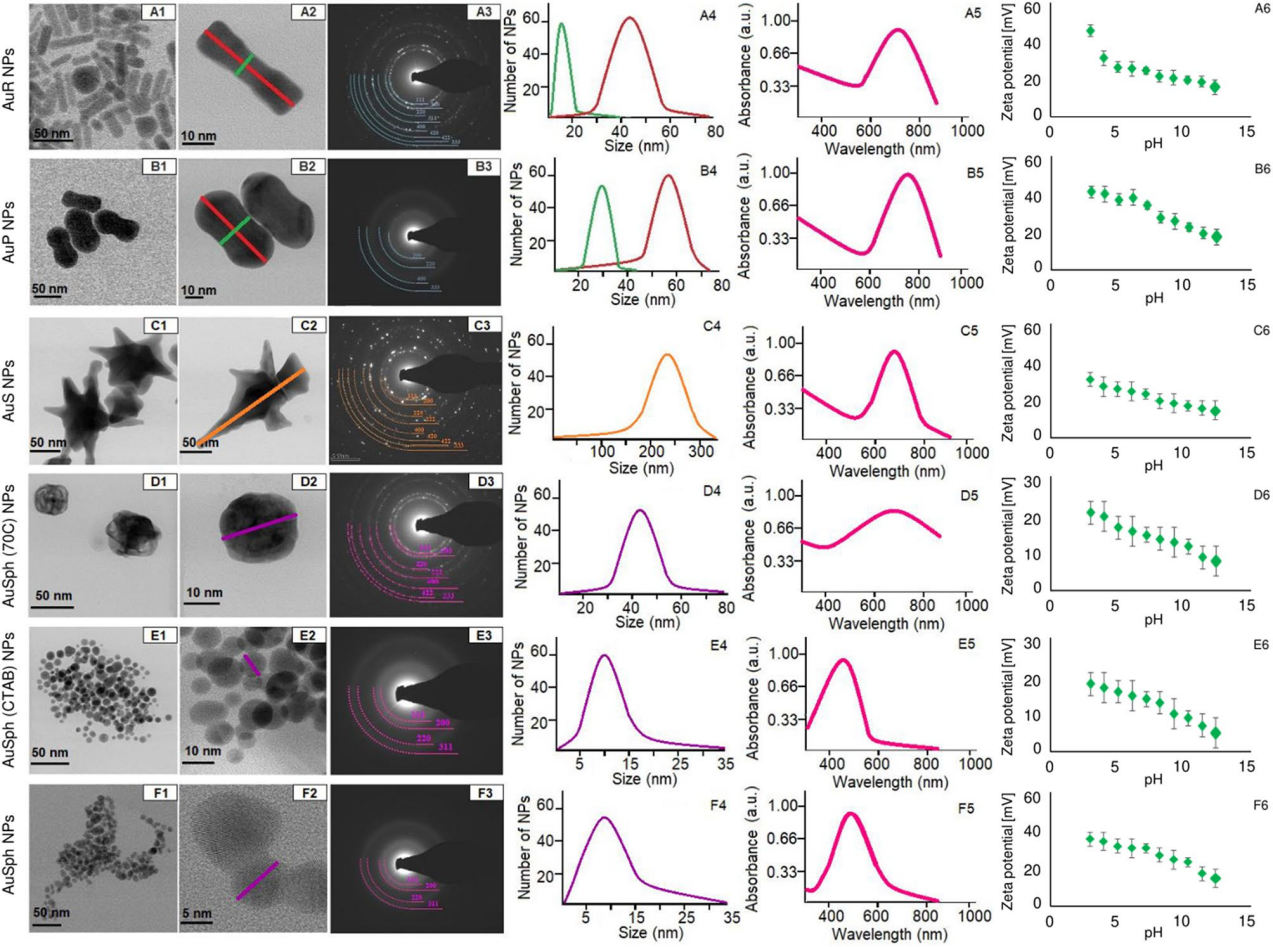
Physicochemical properties of the developed AuR NPs (**A**) AuP NPs (**B**), AuS NPs (**C**), AuSph (70C) NPs (**D**), AuSph (CTAB) NPs (**E**), AuSph NPs (**F**) nanoparticles. Bright field scanning transmission electron microscopy (BF STEM) overview (**A1**–**F1**), magnified images (**A2**–**F2**), and corresponding SAED patterns (**A3**–**F3**) recorded for Au NPs. Size distribution of obtained Au NPs (**A4**–**F4**), UV–Vis spectra of Au NPs (**A5**–**F5**) and zeta potential values of synthesized Au NPs (**A6**–**F6**).

The morphology of the synthesized Au NPs was analyzed by scanning transmission electron microscopy (STEM) (Fig. 1). The nanoparticles synthesized over 0.5 h had an elongated rod-like shape with sharply-rounded ends (AuR NPs) with size of ~ 45 ± 8 nm along the longitudinal axis and ~ 10 ± 3 nm along the transverse axis, Fig. 1A4. In addition, it was noticed that when the transverse axis increased, the longitudinal axis decreased. Extending the synthesis time to 3 h, led to the formation of peanut-like gold nanoparticles with size of ~ 60 ± 5 nm along the longitudinal axis, ~ 25 ± 5 nm along the transverse axis, Fig. 1B4. However, in the STEM images of the AuP NPs it can be seen that the core of the nanoparticles had a rod shape and was surrounded by a gold shell, which caused the shape of the obtained nanoparticles to change their shape to peanut-like. This effect was caused by the CTAB and silver nitrate (AgNO_3_) solutions, which were used up during the synthesis. The generated AgBr–ions block the growth of the AuNPs in the direction^26,27^. Therefore, the nanoparticles were expected to grow parallel to the^16^ planes forming elongated structures as for the AuR NPs in Fig. 1A1,A2. However, it was noted that extending the synthesis time caused the ends of the edges to start growing in the direction, creating the AuP NPs (Fig. 1B1,B2), which was likely due to depletion of the CTAB and the AgNO_3_ particles in the solution at the end of the synthesis^26^. In Fig. 1C1,C2 star-shape AuNPs, which cores have a cube-like shape and 144 nm size, as well as 243 nm at the farthest ends of the stars' arms the diagonal size. These shapes od Au NPs was caused by increasing the reducer concentration. It is responsible for the decrease of time, which was needed for the reduction of chloroauric acid (HAuCl_4_). Furthermore, high reaction speed prevents any reaction between the CTAB and AgNO_3_ solutions and consequently, nanoparticles can growth in non-dominant direction created star arms. Moreover, STEM images of AuSph (70C), Fig. 1D1,D2 showed spherical shape of NPs with sizes around 44 ± 5 nm, but these nanoparticles have pores. These pores were formed as a result of the high temperature synthesis. The condition under high temperature caused increase of the gold precursor reduction speed and unblocking of the nanoparticles growth in the direction by destabilization of CTAB molecules^26^. Moreover, STEM images of AuSph (CTAB) NPs, Fig. 1E1,E2 showed that obtained nanoparticles have spherical shape and average size around 10 nm. Very similar result was obtained for AuSph NPs synthesized using trisodium citrate, Fig. 1F1,F2, which also have spherical shape and around 8 nm size.

To determine the structure of the synthesized nanoparticles, selected area electron diffraction (SAED) patterns from the analyzed nanoparticle samples were acquired (Fig. 1A3–F3). The obtained results revealed that all synthesized Au NPs were crystalline. Moreover, the SAED patterns showed that the randomly oriented obtained gold nanoparticle crystallites could be indexed with the lattice parameter of gold^28^. Generally, the visible rings in SAED appear sharper and contain more and more spots with increasing nanoparticle size. Therefore, the SAED from AuS NPs (Fig. 1C3) have the sharpest rings, while SAED obtained for both spherical Au NPs have fuzzy rings, Fig. 1E3,F3. However, the SAED pattern taken from the AuP NPs was composed of blurred rings, in contrast to the pattern for AuR NPs, which showed visible spots. This may be because the AuP NPs were thicker (Fig. 1B1,B2), particularly at their ends, compared with AuR NPs (Fig. 1A1,A2). Moreover, UV–Vis spectra of Au NPs, showed, that the position of SPR peaks depends on the shape of nanoparticles, Fig. 1A5–F5. For AuR NPs, Fig. 1A5 and AuP NPs, Fig. 1B5, SPR positions were around 768 nm and 775 nm for rod- and peanut-shaped gold nanoparticles, respectively. For AuS NPs, the maximum absorbance was around 680 nm, Fig. 1D5, while porous nanoparticles have SPR peak with wide range between 500 and 900 nm, Fig. 1D5. Furthermore, SPR peaks position for both spherical Au NPs were around 525 nm, Fig. 1E5,F5. To confirm the stability of varied-shaped gold nanoparticles, zeta potential measurements were performed and results of these analyses are presented in Fig. 1A6–F6. Regardless of nanoparticles shape, all synthetized materials were positively charged in pH range 3.5–12.5. Accordingly, for AuR NPs the zeta potential values range from ~ 49 for pH = 3.5 to 18 mV for pH = 12.5, while for AuP NPs, these values are between 42 and 19 mV (Fig. 1A6,B6, respectively). For star-shaped gold nanoparticles zeta potential values range from 33 to 16 mV (Fig. 1C6). Zeta potential values for all spherical gold nanoparticles (Fig. 1D6–F6) are between 35 and 4 mV and depend on the pH solution. For all synthesized nanoparticles the potential value decreases as the pH value increases.

### Developed nanomaterials exert potent bactericidal and fungicidal activity against large spectrum of microbial pathogens

**Figure 2:**
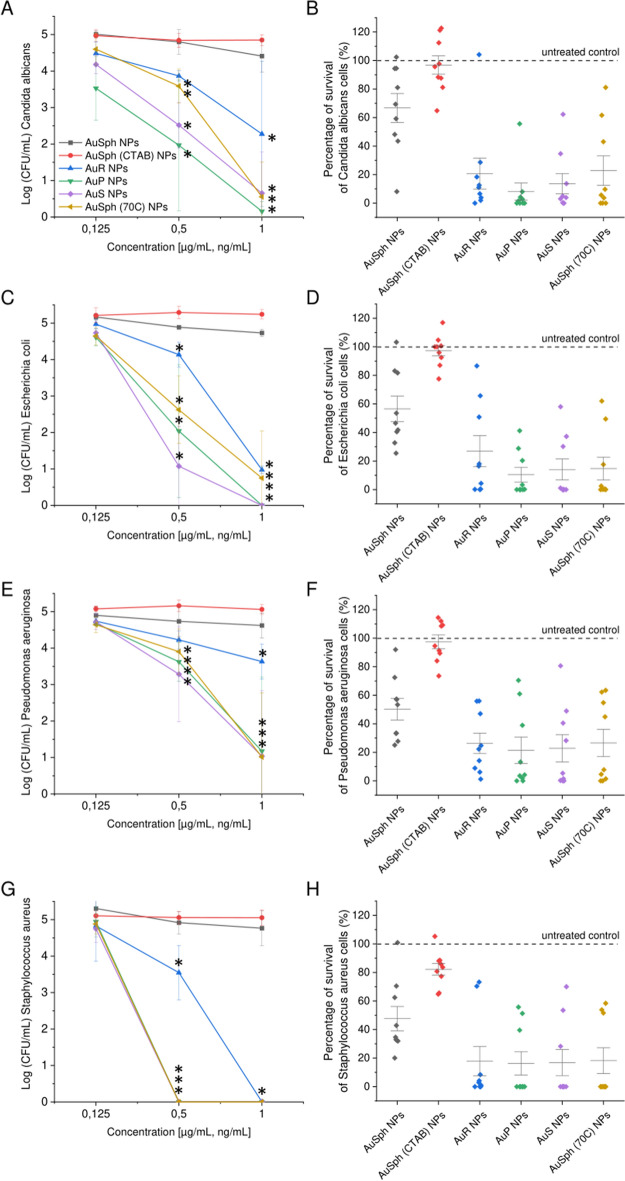
Antimicrobial activity of varied-shaped gold nanoparticles. Killing efficiency of spherical, nonfunctionalized gold nanoparticles (AuSph NPs; black line), CTAB-functionalized spherical nanoparticles [AuSph (CTAB) NPs; red line], gold nanorods (AuR NPs; blue line), gold nanopeanuts (AuP NPs; green line), gold nanostars (AuS NPs; violet line) and porous spherical-shaped gold nanoparticles [AuSph (70C) NPs; gold line] against representative isolates of *C. albicans* [n = 3, (**A**) and (**B**)], *E. coli* [n = 3, (**C**) and (**D**)], *P. aeruginosa* [n = 3, (**E**) and (**F**)] and *S. aureus* [n = 3, (**G**) and (**H**)] was tested using colony counting assay using doses of 0.125, 0.5 and 1 µg mL^−1^ (for AuSph NPs) or 0.125, 0.5 and 1 ng mL^−1^ (for other nanostructures). Results are presented as mean ± SD from 3 representative strains. *Indicates statistical significance (p < 0.05) when compared to untreated bacteria.

Varied-shaped gold nanoparticles were characterized by their antimicrobial activity against representative isolates of *C. albicans* fungi, Gram-negative bacteria from *E. coli* and *Pseudomonas aeruginosa* species, as well as Gram-positive *Staphylococcus aureus* bacteria. As determined using a colony counting assay (Fig. 2) and MIC/MBC/MBIC measurements (Table 1), non-spherical nanoparticles, as well as those porous ones, exert potent fungicidal and bactericidal activity against wild spectrum of microbial pathogens at nanogram doses. Although some variations in susceptibility are recorded among the species and single isolates, a majority of microbial population is noted to be eradicated at concentration of 0.5–1 ng mL^−1^ (Fig. 2). At the same time, no significant effect was observed for both AuSph NPs (despite the employment of doses up to 25 µg mL^−1^) and CTAB-functionalized gold nanospheres, which point out the great potential of shaping morphology of nanomaterials in order to improve their killing activities. When comparing average survival of tested strains, no considerable differences between non-spherical gold nanoparticles were detected, since a median of survival is ranging mostly from 10 to 25% for all tested isolates. Only for *C. albicans* and *E. coli* isolates, AuR NPs were noted to be slightly weaker in microbial eradication, but this effect did not differ statistically (Fig. 2B,D). When comparing the susceptibility of pathogens, *E. coli* and *S. aureus* bacteria were the most prone to non-spherical AuNPs-mediated treatment (Fig. 2B,D).

**Table 1:** Minimal inhibitory concentrations (MIC; ng mL^−1^ or µg mL^−1^), minimal bactericidal/fungicidal concentrations (MBC/MFC; ng mL^−1^ or µg mL^−1^) and minimal biofilm inhibitory concentrations (MBIC; ng mL^−1^ or µg mL^−1^) of varied-shaped gold nanoparticles against *C. albicans, E. coli, P. aeruginosa* and *S. aureus* isolates.

	AuSph NPs (µg mL^−1^)	AuSph (CTAB) NPs (µg mL^−1^)	AuR NPs (ng mL^−1^)	AuP NPs (ng mL^−1^)	AuS NPs (ng mL^−1^)	AuSph (70C) NPs (ng mL^−1^)
*C. albicans*	> 20/> 20/> 20	> 20/> 20/> 20	0.31/0.62/0.62	0.16/0.31/0.31	0.16/0.16/0.31	0.31/0.31/0.62
*C. albicans*	> 20/> 20/> 20	> 20/> 20/> 20	0.62/1.25/1.25	0.62/0.62/0.62	0.31/0.62/0.62	0.62/0.62/0.62
*C. albicans*	> 20/> 20/> 20	> 20/> 20/> 20	0.31/0.62/0.62	0.31/0.31/0.31	0.62/0.62/0.62	0.31/0.62/1.25
*C. albicans*	> 20/> 20/> 20	> 20/> 20/> 20	0.31/0.31/0.31	0.16/0.31/0.31	0.31/0.31/1.25	0.31/0.62/0.62
*C. albicans*	> 20/> 20/> 20	> 20/> 20/> 20	0.62/0.62/1.25	0.62/0.62/0.62	0.31/0.62/0.62	0.62/0.62/1.25
*E. coli*	> 20/> 20/> 20	> 20/> 20/> 20	0.31/0.31/0.62	0.31/0.31/0.62	0.16/0.31/0.31	0.31/0.31/0.62
*E. coli*	> 20/> 20/> 20	> 20/> 20/> 20	0.62/0.62/1.25	0.31/0.31/0.62	0.31/0.62/0.62	0.62/0.62/0.62
*E. coli*	> 20/> 20/> 20	> 20/> 20/> 20	0.31/0.62/0.62	0.31/0.31/0.31	0.31/0.31/0.62	0.31/0.31/0.31
*E. coli*	> 20/> 20/> 20	> 20/> 20/> 20	0.62/0.62/1.25	0.62/0.62/0.62	0.62/0.62/1.25	0.62/1.25/1.25
*E. coli*	> 20/> 20/> 20	> 20/> 20/> 20	0.31/0.31/0.31	0.31/0.62/0.62	0.16/0.31/0.31	0.16/0.62/0.62
*P. aeruginosa*	> 20/> 20/> 20	> 20/> 20/> 20	> 40/> 40/> 40	20/> 40/> 40	40/40/40	40/40/40
*P. aeruginosa*	> 20/> 20/> 20	> 20/> 20/> 20	40/40/> 40	20/20/40	40/40/> 40	20/20/40
*P. aeruginosa*	> 20/> 20/> 20	> 20/> 20/> 20	40/> 40/> 40	20/20/40	40/40/> 40	20/40/40
*P. aeruginosa*	> 20/> 20/> 20	> 20/> 20/> 20	> 40/> 40/> 40	> 40/> 40/> 40	> 40/> 40/> 40	> 40/> 40/> 40
*P. aeruginosa*	> 20/> 20/> 20	> 20/> 20/> 20	20/40/40	> 40/> 40/> 40	40/40/40	40/40/> 40
*S. aureus*	> 20/> 20/> 20	> 20/> 20/> 20	0.08/0.16/0.16	0.08/0.08/0.16	0.08/0.16/0.16	0.08/0.78/0.16
*S. aureus*	> 20/> 20/> 20	> 20/> 20/> 20	0.08/0.08/0.08	0.08/0.16/0.31	0.08/0.31/0.31	0.08/0.16/0.31
*S. aureus*	> 20/> 20/> 20	> 20/> 20/> 20	0.31/0.31/0.62	0.16/0.16/0.31	0.16/0.16/0.62	0.16/0.31/0.62
*S. aureus*	> 20/> 20/> 20	> 20/> 20/> 20	0.08/0.08/0.31	0.08/0.16/0.16	0.08/0.08/0.31	0.08/0.08/0.08
*S. aureus*	> 20/> 20/> 20	> 20/> 20/> 20	0.31/0.31/0.62	0.08/0.16/0.31	0.31/0.31/0.31	0.16/0.31/0.62

High antimicrobial efficiency was also confirmed in the presence of growth medium following 24 h incubation with the tested compounds (Table 1). The broth dilution method revealed that a relatively low dose of nanomaterials, i.e. ranging from 0.78 to 0.625 ng mL^−1^ should be considered as fungicidal and bactericidal, as demonstrated for *C. albicans, E. coli* and *S. aureus* isolates. Importantly, the minimum biofilm inhibitory concentrations were not significantly higher than the bactericidal ones; for the majority of strains the MBIC value was not greater than 0.625 ng mL^−1^. Notably, *P. aeruginosa* isolates were considerably less susceptible to AuNPs-mediated treatment when compared to other strains, since MIC/MBC/MBIC values recorded for 5 representative isolates were ranging from 20 to > 40 ng mL^−1^. It is also consistent with the results of colony counting assay (Fig. 2), presenting lower killing efficiency of non-spherical gold nanoparticles against this species of bacteria. Regardless of this, activity of gold nanorods, nanopeanuts, nanostars and porous gold nanoseeds were recorded to be far more greater than those for spherical-shaped nanoparticles. AuSph NPs and AuSph (CTAB) NPs were ineffective at doses lower than 20 µg mL^−1^ (Table 1).

### Antimicrobial mechanism of developed nanoparticles includes the induction of oxidative stress followed by destruction of pathogen’s membranes and lysis of the cell

**Figure 3:**
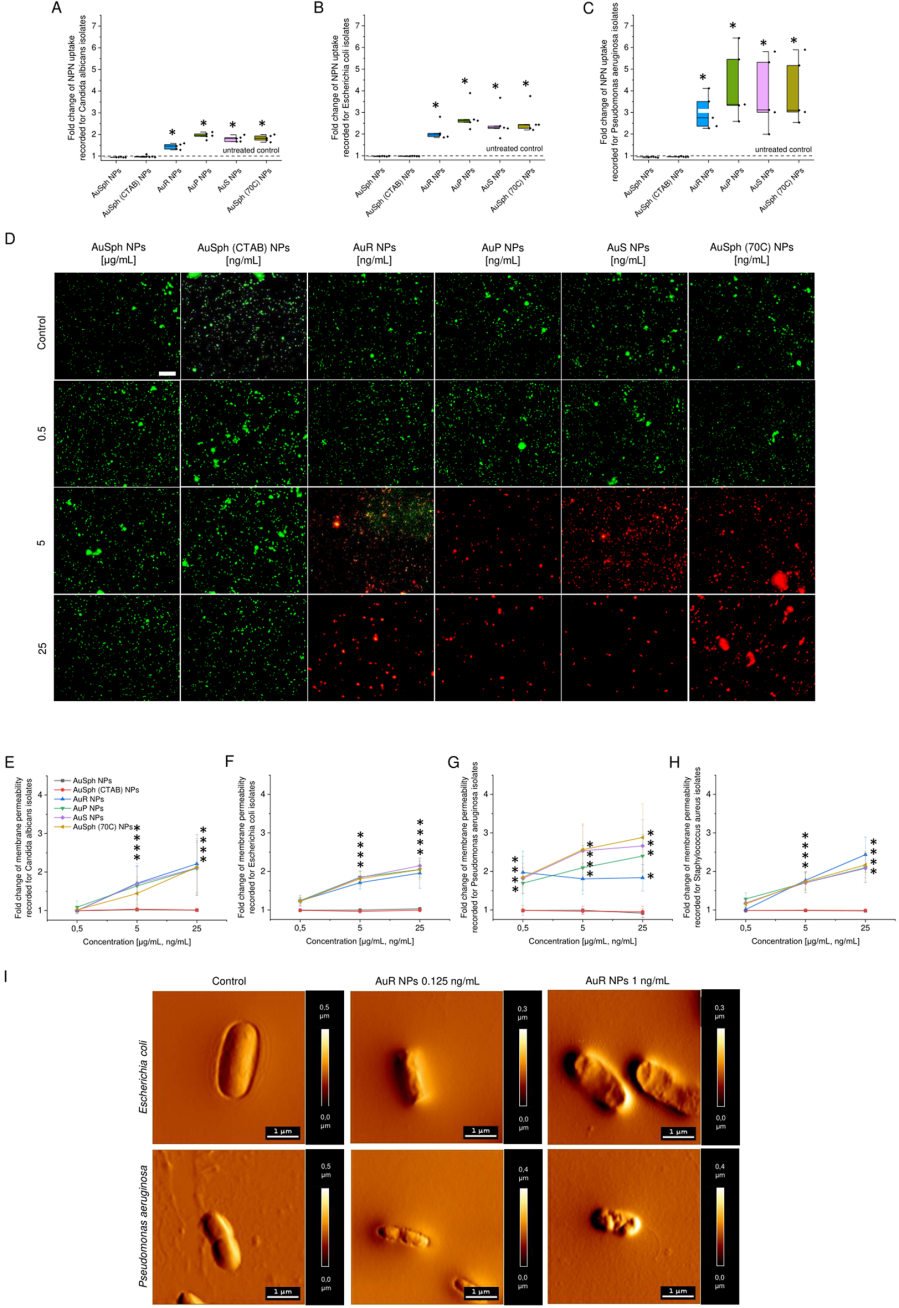
Increase of microbial membrane permeability upon treatment with varied-shaped gold nanoparticles. Uptake of NPN into *C. albicans, E. coli* and *P. aeruginosa* cells induced by treatment with spherical- and non-spherical gold nanoparticles (**A**–**C**). Decrease of viability of *S. aureus* bacteria followed incubation with developed nanoparticles evaluated using fluorescence microscopy (**D**). Scale bar ~ 100 µm. Au NPs-mediated increase of cellular membranes permeability of *C. albicans* (**E**), *E. coli* (**F**), *P. aeruginosa* (**G**) and *S. aureus* cells (**H**). AuR NPs-induced damage of membrane of representative isolates of *E. coli* and *P. aeruginosa* investigated using AFM (**I**). Results are presented as mean ± SD from 5 representative strains (**A**–**C**,**E**–**H**). For panels (**D**) and (**I**), the results from one representative experiment are shown. *Indicates statistical significance (*p* < 0.05) when compared to untreated sample.

The integrity of microbial layers following treatment with the synthesized nanoparticles was investigated using two fluorimetric probes—highly hydrophobic NPN (*N*-phenyl-1-napthylamine) and cell-impermeable PI (propidium iodide). Increase of NPN fluorescence intensity is observed when probe enters the periplasmic space between outer and inner membrane of Gram-negative bacteria, as well as space between fungal cell wall and cell membrane membrane^29,30^. The ability of non-spherical gold nanoparticles to permeate the inner membranes of fungi and bacteria was investigated by analyzing the fluorescence signal recorded when PI binds to DNA of treated microbial cells. Developed nanoparticles cause significant permeability increase in the cell membranes membranes of all tested fungal and bacteria strains, which is evidenced by dose-dependent rise of NPN fluorescence intensity (increase by two- to seven-fold; Fig. 3A–C) and nearly threefold enhancement of PI-derived fluorescence signal when compared with the untreated control microbes (Fig. 3E–H). In contrast to that, spherical-shaped gold nanoparticles, both unfunctionalized (AuSph NPs) and CTAB-functionalized [AuSph (CTAB) NPs] did not affect the membrane architecture of tested microorganisms, which is correlated with their poor killing efficiency. The above observation was confirmed by fluorescence microscopy using SYTO9/propidium iodide (PI)-dual staining, where microbes with intact membranes were stained green and those with impaired ones were indicated by red color. As presented in Fig. 3D, the SYTO9-positive populations of *S. aureus* decreased with increasing concentration of AuR NPs, AuP NPs, AuS NPs and AuSph (70C) NPs, while the population of dead bacteria rose. As expected, viable population of bacteria treated with AuSph NPs at microgram doses did not differ, when compared to control. Similar tendency was observed also for representative strains of *E. coli, P. aeruginosa* and *C. albicans* while analyzing all developed nanoparticles (Supplementary Fig. 3). These results were also confirmed by AFM analysis, which revealed considerable alterations in the morphology of *E. coli* and *P. aeruginosa* bacteria treated with non-spherical nanoparticles. As demonstrated in Fig. 3I, upon exposure to gold nanorods, wrinkles on the surface of the treated bacteria were observed, which was in contrast to the control cells that were characterized by a smooth cell surface. In addition, the decrease in height of the tested samples was recorded, which indicates the decomposition of the cellular structure of microbe. These results confirm our hypothesis that the gold nanoparticles-mediated killing process is determined by membrane-permeabilizing properties. Notably, disruption of both the outer and inner membranes was detected in all tested microbial strains, regardless of the species and the mechanism of drug resistance detected for these isolates, which suggest that developed nanomaterials might be effective against broad spectrum of drug-resistant pathogens.

**Figure 4:**
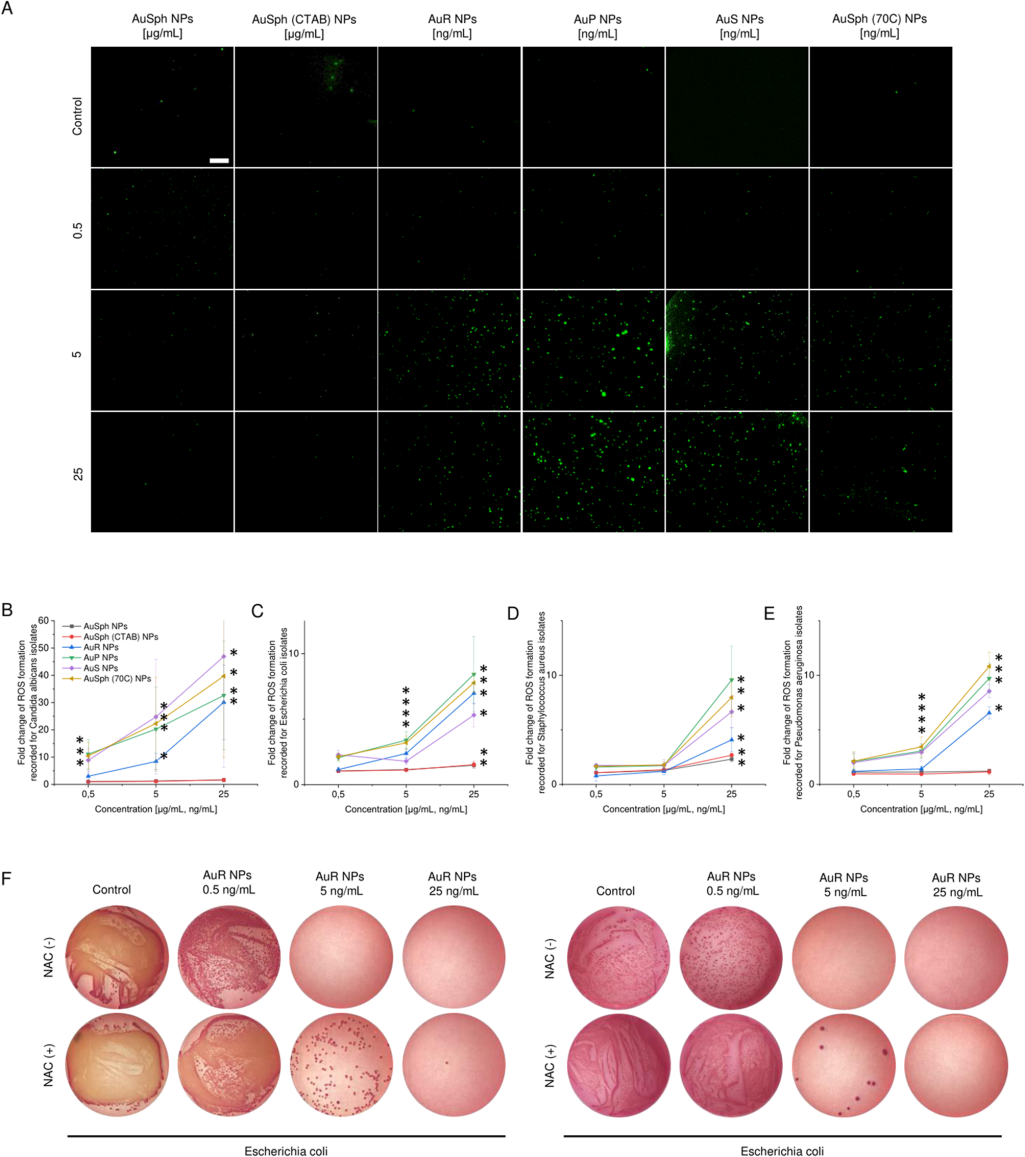
Generation of reactive oxygen species (ROS) in fungi and bacteria subjected to varied-shaped gold nanoparticles over an 1 h incubation. Increase of ROS-positive *E. coli* cells investigated using fluorescence microscopy (**A**). Scale bar ~ 20 µm. Rise in ROS-derived fluorescence signal recorded for *C. albicans* (**B**), *E. coli* (**C**), *P. aeruginosa* (**D**) and *S. aureus* isolates (**E**). Photographs of representative MacConkey agar plates with *E. coli* treated with AuR NPs at the indicated concentrations with or without pre-treatment with NAC for 1 h. (**F**). *Indicates statistical significance (*p* < 0.05) when compared to untreated bacteria. For (**B**)–(**E**) results are presented as mean ± SD from 5 representative strains. For (**A**) and (**F**), the results from one representative experiment are shown. *Indicates statistical significance (*p* < 0.05) when compared to untreated sample.

To investigate whether the killing of fungi and bacteria by the nanoparticles developed in this study is dependent on oxidative damage-associated pathways, ROS generation upon AuNPs treatment was measured using 2,7-dichlorofluorescin-diacetate (DCFH-DA) as a fluorescent indicator of intracellular radicals. As shown in Fig. 4A, when *E. coli* were cultivated without test agents, only a residual fluorescent signal was detected, while a strong signal derived from DFCH-DA fluorescent probe was measured after treatment with increasing concentrations of non-spherical nanoparticles and those porous ones. No considerable effect was observed when bacteria were treated with AuSph NPs and AuSph (70C) NPs. Since analogous effect was observed for other pathogens as well (Supplementary Fig. 4), we hypothesized that gold nanoparticle-induced ROS generation is responsible for their antimicrobial effect. Quantitative analysis have shown that treatment with non-spherical gold nanoparticles resulted in rapid augmentation of ROS levels compared with the untreated control, which indicates that the tested agents primarily kill microbes via ROS production (Fig. 4B–E). Moreover, this free radical burst was strongly correlated with increases in membrane permeability and the antibacterial activity of the developed nanomaterials (Figs. 2, 3). This indicates that disrupting the membrane integrity through the generation of intracellular ROS is the most like mechanism for the antimicrobial activity of non-spherical gold nanoparticles and porous, spherical ones.

Moreover, to confirm the involvement of ROS in the bactericidal mechanism of non-spherical gold nanoparticles, the efficiency of AuR NPs against *E. coli* was evaluated in the presence of *N-*acetyl-cysteine (NAC). The data visualized in Fig. 4 show that the killing efficacy of the gold nanoparticles was significantly attenuated by the presence of antioxidant. Taken together, the collected data indicate that the superior antimicrobial activity of the developed nanoparticles is determined by ROS formation followed by membrane damage causing pathogens’ death.

### Developed nanoparticles are highly effective against uropathogenic *E. coli* and strongly limit the formation of biofilm on the surface of latex urinary catheters

Considering the potent antimicrobial activity of synthetized non-spherical gold nanoparticles presented in Figs. 2, 3, 4 and given our previous study, demonstrating anti-adhesive properties of gold nanorods^31^, we hypothesized that developed nanomaterials might be successfully used as biomedical devices coatings. Since tested nanoparticles were highly effective against representative *E. coli* isolates used in the previous stage of research, we decided to explore the potential of rod- and peanut-shaped gold nanoparticles as coatings for urinary catheters, which are generally made from materials that allow colonization of uropathogenic bacteria. For this purpose, we expanded our study on 10 clinical strains of *E. coli* isolated from the patients with urinary tract infections with internal numbers from 164/2 to 837. Strains used in this part of the study were isolated from subjects aged from 6 months to 84 years, 3 males and 7 females, with significant bacteriuria ranging from 10^4^ to > 10^7^ CFU/mL (Supplementary Table 1).

The antibiotics sensitivity of the tested strains was variable (Supplementary Table 2), showing a lower drug resistance ratio for children than adults, for which drug resistance was noted for at least 4 conventional antibiotics. More importantly, 6 and 7 of the 10 *E. coli* isolates were resistant to two antibiotics commonly used to treat UTIs—ciprofloxacin and trimethoprim/sulfamethoxazole, respectively. Furthermore, two of these isolates (*E. coli* 369 and *E. coli* 419) were also classified as ESBL (extended-spectrum β-lactamase) positive. Potent therapeutic efficiency was noted for gentamicin (9 strains sensitive, 1 strain intermediate), amikacin (10 strains sensitive), and nitrofurantoin (9 strains sensitive, 1 strain resistant). Based on these findings, the *E. coli* strains were divided into ESBL-positive, ciprofloxacin-resistant, and ciprofloxacin-sensitive groups for the testing and analysis in this research.

The bactericidal activity of rod- and peanut-shaped gold nanoparticles against isolates of *E. coli* was determined using a colony counting assay. As presented in Supplementary Fig. 1, both AuP NPs and AuR NPs exhibited potent bactericidal effects at low nanoparticle concentrations i.e. 0.125–0.5 ng mL^−1^, and gold nanoparticles at a dose of 1 ng mL^−1^ were recognized as bactericidal for nearly all of the tested strains (Supplementary Fig. 1A,F). For the lowest tested dose, 0.125 ng mL^−1^, the average survival rates over all of the tested stains were 42.68 ± 23.20% and 36.08 ± 20.82% for AuP NPs and AuR NPs, respectively, indicating their killing ability even at low doses (Supplementary Fig. 1G). Notably, no considerable differences in bactericidal efficiency were noted between ESBL-positive, ciprofloxacin-resistant, and ciprofloxacin-sensitive strains. Statistical analysis of the differences in the killing abilities of AuP NPs and AuR NPs performed by comparing 30 survival points for each nanoparticle type, also showed no significant differences in the killing properties of the materials (average survival after analysis of all doses was 18.12 ± 24.15% for AuP NPs and 17.69 ± 20.64% for AuR NPs) (Supplementary Fig. 1H).

Both rod- and peanut-shaped gold nanoparticles exerted also high membrane activity as evidenced by β-galactosidase release from the treated bacteria cells. When the bacterial membrane is intact, β-galactosidase is localized intracellularly, however when the inner bacterial membrane is compromised β-galactosidase leaks out, allowing enzymatic hydrolysis of 2-nitrophenyl β-d-galactopyranoside (ONPG) to o-nitrophenol (ONP), which can be spectrophotometrically recorded at 420 nm. As demonstrated in Supplementary Fig. 2, nearly fourfold enhancement of β-galactosidase activity when compared with the untreated control bacteria was recorded. These data confirm the previous results demonstrating potent killing activity of non-spherical gold nanoparticles.

**Figure 5:**
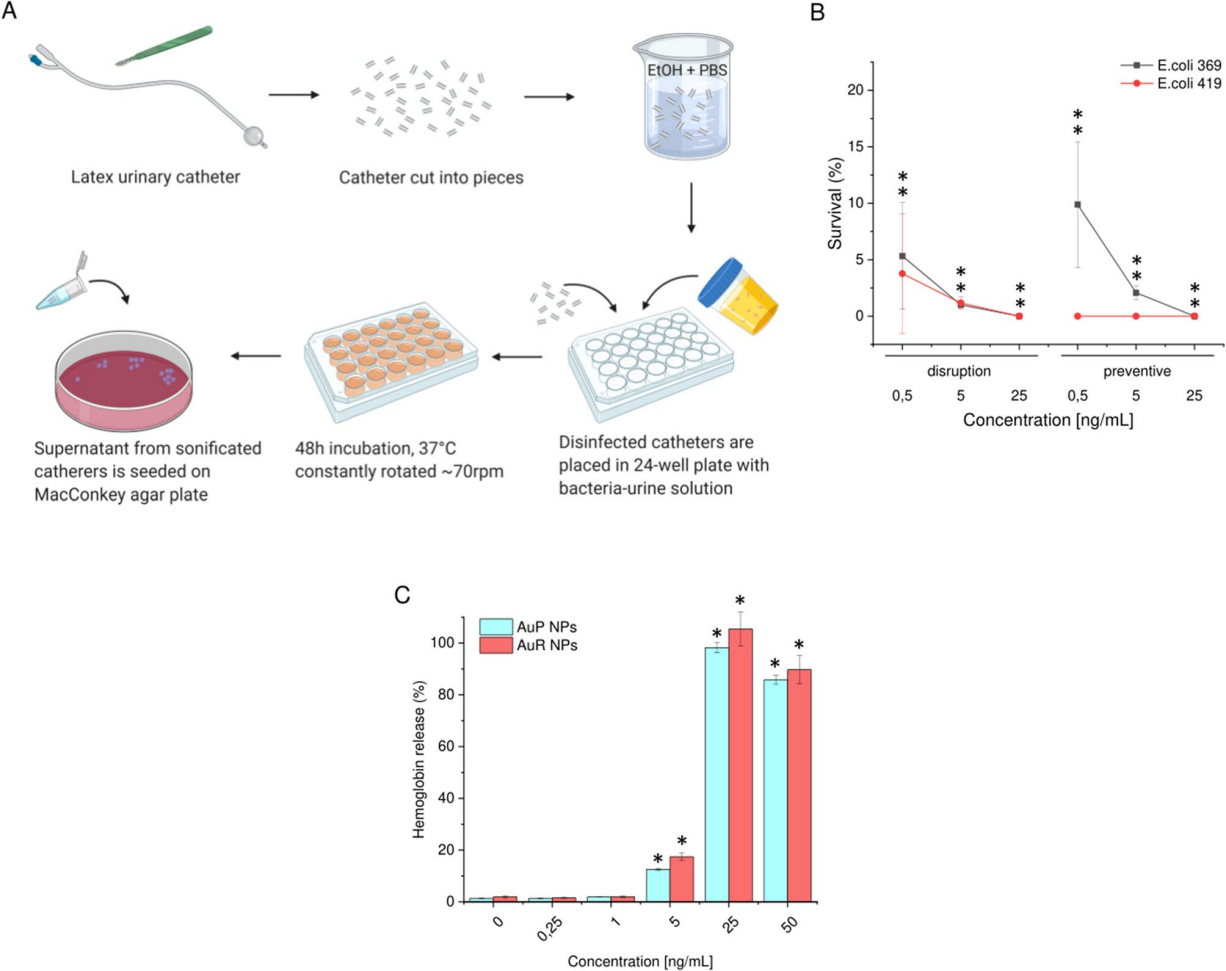
Overview of the urinary catheters-test experiment (**A**). Prevention of *E. coli* attachment and viability on latex urinary catheters incubated in the presence of bacteria-inoculated urine for 48 h (**B**). The release of hemoglobin from damaged RBCs upon treatment with AuP NPs and AuR NPs was investigated using a hemolysis assay (**C**). *Indicates statistical significance (*p* < 0.05) when compared to erythrocytes incubated in the presence of PBS. Figure presented in (**A**) was created with BioRender.com.

Using a simplified method to preliminarily quantify the viability of bacteria on the surface of the urinary catheters, we demonstrated the ability of nanorods and nanopeanuts to limit bacterial attachment to a material that is typically quite easily colonized by uropathogenic bacteria. A summary of the test is presented in Fig. 5A. The tested nanomaterials were highly effective for inhibiting bacteria attachment, proliferation, and survival on the surface of catheters. Compared with untreated latex catheters, biofilm formation by *E. coli* 369 was limited to 9.87 ± 5.57% at a AuR NP concentration of 0.5 ng mL^−1^; and at a dose of 25 ng mL^−1^ no bacterial growth was detected. In addition, the *E. coli* 419 strain was completely inhibited and eradicated even at the lowest dose of material. The lowest dose of AuR NPs was also able to disrupt biofilms pre-formed on the surface of urinary catheter. At a dose of 0.5 ng mL^−1^ the biofilms formed by *E. coli* 369 and *E. coli* 419 were disrupted to a value of 5.33 ± 4.71% and 3.76 ± 5.32%, which further indicated the high potential of non-spherical gold nanoparticles for use in coatings for medical devices, including urinary catheters (Fig. 5B).

### Rod- and peanut-shaped gold nanoparticles exert high biocompatibility at bactericidal doses

Above assumptions on potential utility of AuR NPs and AuP NPs as materials coatings were further supported by the satisfactory biocompatibility of the nanoagents in the bactericidal concentration range. Owing to the high membrane-permeabilizing properties of the non-spherical gold nanoparticles, a biocompatibility test based on hemoglobin release from damaged erythrocytes (hemolysis assay) was performed. As presented in Fig. 5C, damage of erythrocytes membranes was recorded when a dose of AuP NPs or AuR NPs of at least of 25 ng mL^−1^ was applied, which provides indication that in bactericidal doses (i.e. ~ 1 ng mL^−1^), the synthesized nanoagents should be recognized as safe for human cells.

### Both AuP NPs and AuR NPs maintain their killing activity in the presence of urine and variable pH

For a material to be designed as a coating for biomedical devices, it is necessary to ensure that it will be effective in the physiological settings in which the device will be expected to perform. Since we propose that the rod- and peanut-shaped gold nanoparticles will be suitable as urinary catheter coatings, we made an attempt to verify that nanoparticles do not lose their activity in the presence of urine and variable pH. Notably, composition of urine and variable urinary pH affect the growth of uropathogens, and considerably alter the antimicrobial activity of some conventional antibiotics^32,33^. To test whether changes in urinary pH affect the killing abilities of the developed nanoparticles, minimum bactericidal doses were measured in growth medium adjusted to pH 5, 7, and 9 (Table 2 and Supplementary Table 3). As shown, at pH 5 the gold nanoparticles maintained their killing activity in 40% of the bacterial strains. The MBC values increased for 5/10 strains, nevertheless, they did not exceed 0.625 ng mL^−1^. In alkaline environments a considerable decrease in the MBC values from 0.32 to 0.08 ng mL^−1^ and thus an improvement of the therapeutic efficiency was noted, suggesting the synthesized nanoparticles have potential not only against *E. coli*, but also urease-producing bacteria such as *P. aeruginosa* and *P. mirabilis*^34^. This behavior was very similar to that of ciprofloxacin, but more favorable than those of gentamicin, tobramycin, and doxycycline.

**Table 2:**
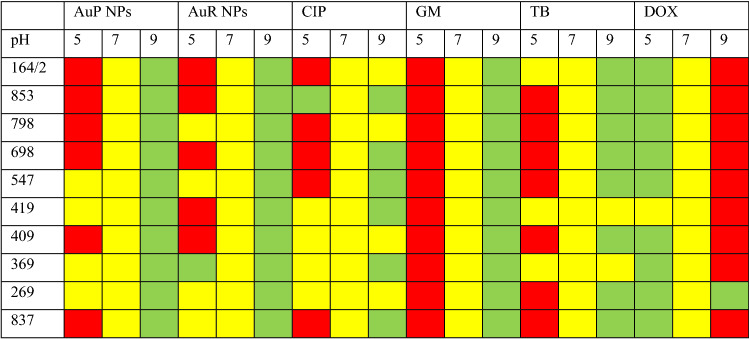
Alterations in peanut- and rod-shaped gold nanoparticles killing activity when tested in growth medium with different pH values compared to conventional antibiotics. Graph was constructed by comparing MIC values recorded for indicated clinical isolates of *E. coli* in LB adjusted to 5, 7 or 9 pH. MIC value in LB (pH = 7; yellow color) was the reference point for further comparisons. Red and green color indicate the rise or drop in MIC value, respectively.

*CIP* ciprofloxacin, *GM* gentamicin, *TB* tobramycin, *DOX* doxycycline.

**Figure 6:**
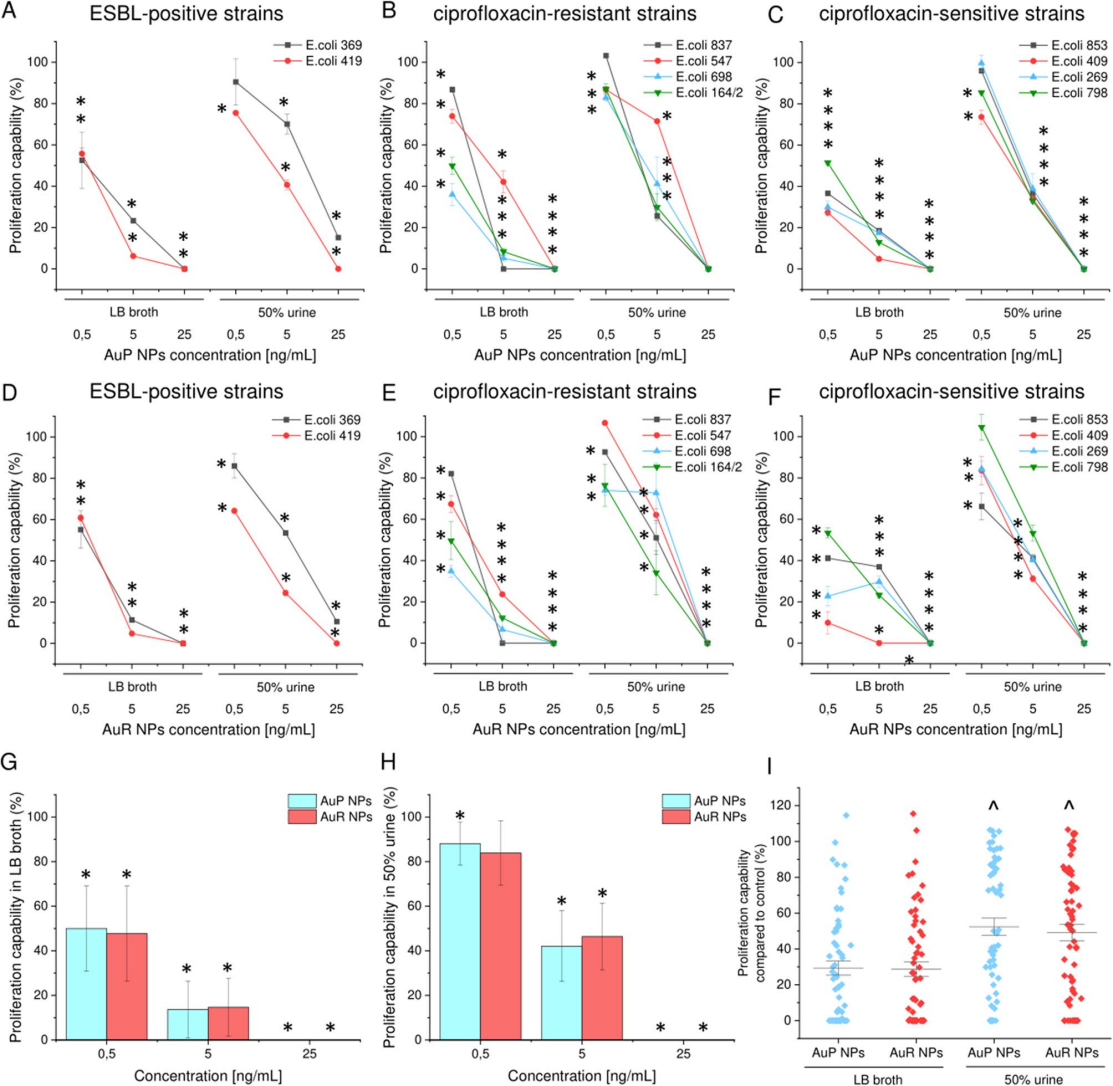
Restriction of *Escherichia coli* proliferation upon treatment with peanut- (AuP NPs; **A**–**C**) and rod-shaped gold nanoparticles (AuR NPs; **D**–**F**) in the presence of LB broth or LB broth with 50% urine. The proliferation of bacteria upon treatment with gold nanoparticles with concentrations of 0.5, 5, and 25 ng mL^−1^ was tested using a resazurin-based fluorimetric assay against ESBL-positive strains [(**A**) and (**D**); *E. coli* 369 and 419], ciprofloxacin-resistant strains [(**B**) and (**E**); *E. coli* 837, 547, 698, and 164/2], and ciprofloxacin-sensitive strains [(**C**) and (**F**); *E. coli* 853, 409, 269, and 798]. Panels (**G**) and (**H**) demonstrate the restriction of proliferation of 10 UTI-associated *E. coli* strains in LB (**G**) and LB containing 50% urine (**H**) as average values. Statistical analysis of the differences in the anti-proliferative abilities of AuP NPs and AuR NPs, in the presence of both growth medium and medium containing 50% urine is presented in panel (**I**). Results are presented as mean ± SD for 3–20 measurements. * and ^ indicates statistical significance (*p* < 0.05) when compared to untreated bacteria and samples incubated in LB broth, respectively.

Additionally, in order to introduce the developed nanoparticles into clinical settings, an appropriate antimicrobial efficacy in the presence of human urine must be achieved. The complex composition of human urine, which includes a variety of organic and inorganic ions, not only supports bacteria growth, but can also affect the solubility and stability of antimicrobials. To determine the impact of human urine on the killing properties of AuP NPs and AuR NPs, a fluorimetric-based viability assay was performed and the proliferation of bacterial strains in the presence of LB or LB supplemented with 50% urine was compared. As shown in Fig. 6A–F, some increases in proliferation were noted for bacteria cultured in the presence of human urine; the metabolic activity and survival of *E. coli* bacteria treated with the lowest dose of AuP NPs and AuR NPs, i.e. 0.5 ng mL^−1^, were approximately 1.75-fold higher than those of bacteria cultivated in growth medium only. Significant differences were also noted for samples treated with 5 ng mL^−1^ of nanoparticles (increases from 13.65 ± 12.56% and 14.68 ± 13.06% to 42.10 ± 15.84% and 46.38 ± 14.86% for AuP NPs and AuR NPs, respectively). Under the same experimental conditions, nanoparticles at a dose of 25 ng mL^−1^ exerted bactericidal effects regardless of the addition of human urine (Fig. 6G–H). The killing activity of peanut-shaped gold nanoparticles appeared to be less affected by the addition of urine than rod-shaped particles, however no statistical differences between AuP NPs and AuR NPs were noted (Fig. 6I).

### AuP NPs and AuR NPs exert potent anti-biofilm activity

**Figure 7:**
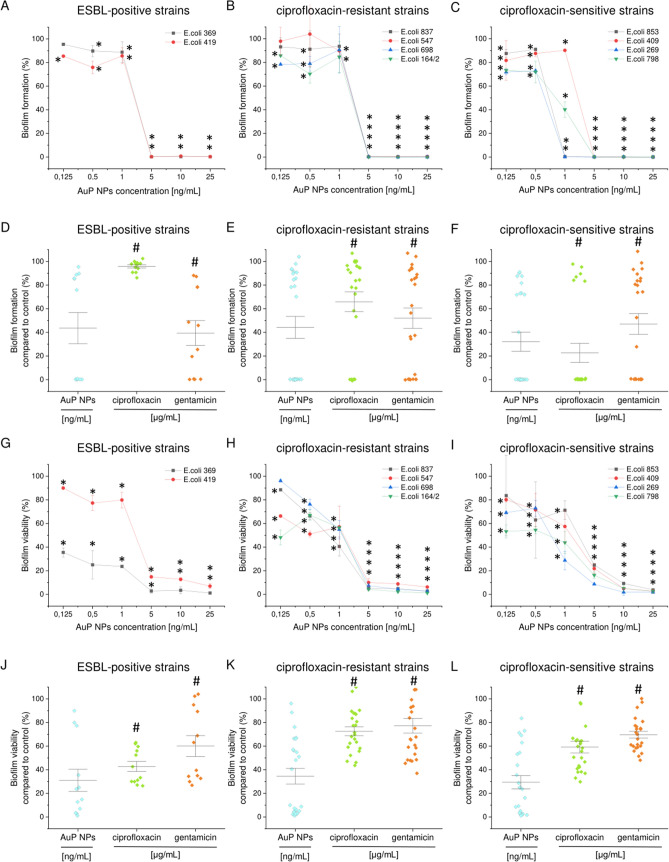
Prevention of biofilm formation by clinical isolates of *E. coli* (**A**–**F**) and disruption of pre-formed biofilms (**G**–**L**) by peanut-shaped gold nanoparticles (AuP NPs) compared with ciprofloxacin and gentamicin. The formation of biofilms and viability of disrupted biofilms was assessed using a resazurin-based fluorimetric method after 48 h of cultivating against ESBL-positive strains (**A**,**D**,**G**,**J**; *E. coli* 369 and 419), ciprofloxacin-resistant strains (**B**,**E**,**H**,**K**; *E. coli* 837, 547, 698 and 164/2), and ciprofloxacin-sensitive strains (**C**,**F**,**I**,**L**; *E. coli* 853, 409, 269 and 798). Panels (**D**–**F**) and (**J**–**L**) compare the anti-biofilm activities of AuP NPs, ciprofloxacin, and gentamicin in doses ranging from 0.125 to 25 ng mL^−1^ (for AuP NPs) and 0.125 to 25 µg mL^−1^ (for ciprofloxacin and gentamicin). * and # indicate statistical significance (*p* < 0.05) when compared to untreated control samples and nanoparticles-treated bacteria, respectively.

**Figure 8:**
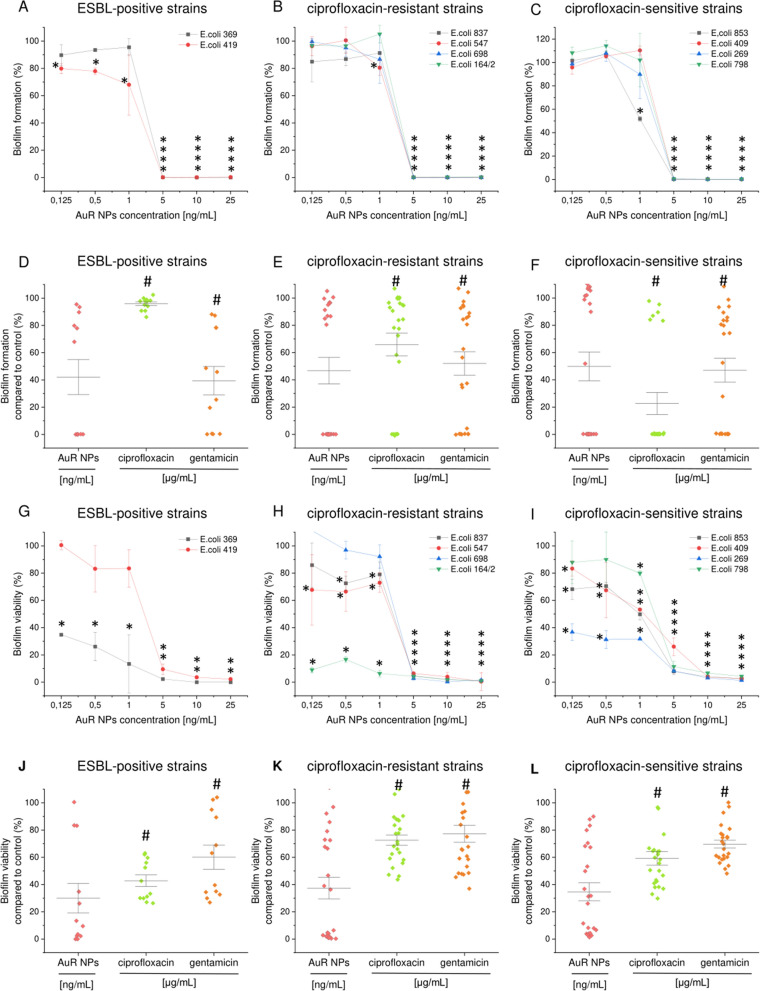
Prevention of biofilm formation by clinical isolates of *E. coli* (**A**–**F**) and disruption of pre-formed biofilms (**G**–**L**) by rod-shaped gold nanoparticles (AuR NPs) compared with ciprofloxacin and gentamicin. The formation of biofilms and viability of disrupted biofilms was assessed using resazurin-based fluorimetric methods after 48 h of cultivating against ESBL-positive (**A**,**D**,**G**,**J**; *E. coli* 369 and 419), ciprofloxacin-resistant (**B**,**E**,**H**,**K**; *E. coli* 837, 547, 698 and 164/2), and ciprofloxacin-sensitive (**C**,**F**,**I**,**L**; *E. coli* 853, 409, 269 and 798) strains. Panels (**D**–**F**) and (**J**–**L**) compare the anti-biofilm activities of AuR NPs, ciprofloxacin, and gentamycin in doses ranging from 0.125 to 25 ng mL^−1^ (for AuR NPs) and 0.125 to 25 µg mL^−1^ (for ciprofloxacin and gentamycin). * and # indicate statistical significance (*p* < 0.05) when compared to untreated control samples and nanoparticles-treated bacteria, respectively.

Biofilms are communities of surface-attached microorganisms embedded in a polymeric matrix consisting of self-produced substances, which protect the bacteria against chemical and mechanical stress factors and increase their resistance to applied antibiotics^35^. The production of biofilms is recognized as a crucial determinant of long-term persistence of bacteria in the urinary tract and a factor determining recurrence of urinary tract infections^36^. In effect, targeting biofilms might offer an opportunity to control biofilm infections^35,37^. In this study, to assess the anti-biofilm properties of the nanomaterials, we used resazurin biofilm staining, which is based on the ability of metabolic enzymes in the cytoplasm of viable bacteria to convert rezasurin into highly fluorescent resofurin^38^. The rezasurin viability assay is considered more accurate than conventional biofilm-counting methods^39^. Two approaches were used to investigate the anti-biofilm properties of the developed nanoparticles: in the first, the preventive properties against *E. coli*-formed biofilms were assessed (Figs. 7A–F, 8A–F), in the second, the ability of AuP NPs and AuR NPs to disrupt pre-formed biofilms were determined (Figs. 7G–L, 8G–L). The peanut- and rod-shaped nanoparticles were highly effective in inhibiting biofilm formation. At a dose of 5 ng mL^−1^ no viable biofilm-embedded cells were detected in any of the tested isolates. When averaged, AuP NPs and AuR NPs were far more effective than the conventional antibiotics ciprofloxacin and gentamicin, even when the antibiotics were used at 1000 times higher doses (i.e. 0.125–25 µg mL^−1^).

When the survival points for all of the tested doses were compared, the biofilm formation by ESBL-positive, ciprofloxacin-resistant, and ciprofloxacin-sensitive strains was inhibited to 43.55%, 44.18%, and 32.07% (for peanut-shaped gold nanoparticles) and 42.08%, 46.71%, and 49.82% (for rod-shaped nanomaterials), respectively. In addition, ciprofloxacin and gentamicin were much less effective as 1000-fold doses were required to achieve poorer results (Figs. 7D–F, 8D–F). Notably, further anti-biofilm performance was observed when the synthesized nanoparticles were tested as biofilm disrupting agents. Compared with the untreated pre-formed *E. coli* biofilms, AuP NPs and AuR NPs were able to significantly disrupt the tested biofilms even at low nanomaterial doses. The disruption ability of the developed nanoparticles at a dose of 0.125 ng mL^−1^ ranged from 35.52 ± 3.83% to 96.11 ± 1.04% (71.07 ± 20.17% on average) and from 34.79 ± 0.68% to 111.15 ± 33.26% (68.52 ± 32.36% on average) survival for AuP NPs and AuR NPs, respectively. Doses of 5–10 ng mL^−1^ were found to completely disrupt pre-formed established biofilms of *E. coli* (Figs. 7G–I, 8G–I). Ciprofloxacin and gentamicin were less effective against all of the tested strains, even when used at greater than 1000-fold higher concentration (Figs. 7J–L, 8J–L). Notably, the developed nanoparticles were more effective at disrupting the established bacterial biofilms than preventing the formation of new ones, which suggests that AuP NPs and AuR NPs possess some ability to interact with/and penetrate the extracellular matrix in which the bacteria are embedded, and breaking down the biofilm matrix makes them more prone to nanoparticle-mediated treatment.

## Discussion

The antimicrobial effects of metal nanoparticles are a result of their morphology and surface charge, which are the critical factors that affect the interaction of NPs with microorganisms^40–42^. AuNPs have therefore been synthesized in a broad spectrum of sizes and shapes, including spherical nanoparticles^22,43^, nanorods^22^, nanocubes^26^, nanostars, and nanoflowers^25^. In our study, through the addition of CTAB—a cationic agent commonly used in the synthesis of varied-shape nanomaterials^44^—and by varying the synthesis time, we developed a series of varied-shaped gold nanoparticles in shape of nanorods (AuR NPs), nanopeanuts (AuP NPs) and nanostars (AuS NPs), as well as spherical-like porous nanoparticles [AuSph (70C) NPs]. For the purpose of additional controls, unfunctionalized gold nanoseeds (AuSph NPs) and CTAB-functionalized gold nanoparticles [AuSph (CTAB) NPs] were prepared. Modifications of synthesis methods allowed us to obtain nanomaterials, which are characterized by potent fungicidal and bactericidal activity against fungal strains, Gram-negative and Gram-positive bacteria at doses unreported before as low 0.5–1 ng mL^−1^ (Fig. 2, Table 1). In the majority of previous studies, the killing activity of gold nanoparticles was achieved in concentrations ranging from 5 to 512 µg mL^−1^^22,25,43^, i.e., thousands-fold higher doses than those reported in this study. Two potential explanations for the superior bactericidal activity are: (i) “a side effect” of CTAB, used as a shape-directing agent during the synthesis of the nanoparticles or (ii) a unique physicochemical feature of the nanoparticles themselves. Some reports suggest that CTAB might determine the bactericidal efficiency of nanorods against *Staphylococcus aureus* and *Cutibacterium acnes* and induce a superoxide stress in *E. coli*^45,46^. In this context, the possibility that gold nanoparticles could serve as a drug delivery system for the CTAB could be considered, however, the experimental data obtained exclude this possibility. In our experimental protocol we washed the CTAB out of the AuNP reaction mixture after finalizing the synthesis, and the amount of CTAB left on the surface of the nanorods and nanopeanuts was determined by FT-Raman spectroscopy (Supplementary Fig. 5). The calculated percentage of CTAB remaining in the nanoparticle solutions after rinsing was 6% and 10% for the AuP NP and AuR NP solutions, respectively (Supplementary Fig. 3). Since the bactericidal doses of the tested nanoparticles were below 1 ng mL^−1^, it can be concluded that the concentration of CTAB would be as low as 60–100 pg mL^−1^, which, to our knowledge, is unlikely to exert such potent killing behavior.

This conclusion was supported by the results of additional experiments intended to estimate the bactericidal activity of CTAB against the tested fungal and bacterial strains. The MIC/MBC values of CTAB were much higher than those reported for the developed nanoparticles (> 32 µg mL^−1^ vs. 0.078–40 ng mL^−1^ for CTAB and AuNPs nanoparticles, respectively; data not shown). More importantly, we did not observe any considerable effect of CTAB-functionalized spherical gold nanoparticles [AuSph (CTAB) NPs], which confirms that the presence or CTAB on the surface of gold nanoparticles is not a main determinant of their killing ability. Likewise, the observation of Castillo-Martínez et al. that gold nanorods fabricated in the presence CTAB are bactericidal for *E. coli* at a concentration of ~ 7 µg mL^−1^, does not indicate that CTAB markedly improved the bactericidal activity of the final product in this study^47^. In contrast, the second hypothesis linking the superior bactericidal effect to morphological futures of the AuNPs is supported by a compelling number of reports. Non-spherical nanomaterials that feature sharp edges or protrusions with a high aspect ratio may cause high local stress on the bacterial cells causing membrane rupture^48,49^. Similarly, the increased surface area to volume ratio of non-spherical nanoparticles and their enhanced adsorption and binding of compounds, as well as increased ROS generation are considered crucial factors for their biological activity^22,26,41^. For instance, the abrasiveness of the surface of zinc oxide nanoparticles was demonstrated to determine their enhanced antibacterial effect^50^. Likewise, better antibacterial activity has been reported for graphene nanowalls compared with graphene nanosheets owing to the sharper edges of the nanosheets, which considerably compromised cellular membrane and increased membrane permeability and leakage of intracellular material^48^. Therefore, we suggest that a similar type of shape-dependent interaction resulting in an increased ability of nanoparticles to interact with the microorganism is the basis for the superior bactericidal activity of the nanoparticles developed in this research. Since we do not observe a significant differences between killing efficiency of non-spherical gold nanoparticles, we believe that inducting of local stress on bacteria membrane and increasing the surface of contact between bacteria and nanoparticle is far more important than developing a specific shape of nanoparticle by itself. However, it is possible that shape and size of developed nanoparticles will affect the behaviors other than killing bacteria, such as activity in highly viscous body fluids (e.g. pus) or distribution in biofilms. This issue requires however more experimental work.

Previous studies have reported the mechanisms by which gold nanoparticles exert bactericidal effects against bacteria^23,51^. For instance, Ortiz-Benitez et al. demonstrated that gold nanoparticles promote lysis of *Streptococcus pneumoniae* as a result of recruitment of lipids, proteins, and carbohydrates, which leads to pore formation and subsequent cell damage^24^. Furthermore, Cui et al*.* demonstrated that AuNPs in *E. coli* induce membrane potential collapse, and inhibit ATPase activity, or in another reported pathway, inhibit a subunit of ribosome from binding tRNA^51^. In contrast, Lee et al. revealed that the mechanism of AuNP activity against *E. coli* involves DNA damage through apoptosis-like pathways^23^. In our study, we demonstrated that non-spherical gold nanoparticles exert bactericidal effects through formation of reactive oxygen species followed by permeabilization of the outer and inner bacterial membranes, which ultimately led to bacterial death (Figs. 3, 4, Supplementary Fig. 2). Interestingly, in previously published reports the mechanism of AuNP-mediated killing was independent of reactive oxygen species formation, which is in contrast to data acquired in this research^52^. Nevertheless, it should be noted that in both of the reports referenced spherical gold nanoparticles were used, in contrast to the non-spherical ones used in this research. Although the effect of residual CTAB on the surface of the developed nanoparticles cannot be excluded entirely^46^, it should be noted that a number of studies indicate ROS-promoting killing mechanisms for gold nanoparticles against microbial pathogens, including methicillin-resistant *Staphylococcus aureus*^53,54^ and *Corynebacterium pseudotuberculosis*^55^. Notably, in research by Nasser et al., positively-charged gold nanorods were more efficient in ROS triggering than spherical nanogold. It was demonstrated that the increased surface area and higher surface area to charge ratio of the nanorods promoted further ROS production^56^. We believe that a similar phenomenon occurs under our experimental conditions and the ROS-promoting properties are determined by the non-spherical shape of the nanomaterials, which contributes to increased local stress on the bacterial wall followed by ROS production and bacteria killing.

A potential application of the nanoparticles developed in this study is as antimicrobial coatings for urinary catheters, which are generally made from materials that allow colonization by fungi and uropathogenic bacteria. Although some examples of silver NP-containing antimicrobial catheters have been demonstrated to date, there remains a need to improve their effectiveness. Only bactericidal, biocompatible, and anti-adherence agents are therefore considered as coating materials for medical devices. Despite numerous agents currently being tested as antimicrobial coatings^57^, the data on gold-coated urinary catheters remains very limited and gold is not utilized in a single medical device coating. The main factors contributing to the persistence and recurrence of urinary tract infections include (i) drug resistance, (ii) biofilm formation, (iii) chronic inflammatory states, and (iv) attachment of bacteria to medical devices. Based on the presented data, we believe that employment of non-spherical gold nanoparticles might successfully address these issues. Firstly, they are characterized by potent antibacterial activity against broad spectrum of pathogens, including *E. coli* bacteria (Fig. 2, Supplementary Fig. 1), which is recognized as a primary cause of catheter-associated urinary tract infections in hospital and in outpatients^58^. Crucially, this effect was maintained in the presence of urine (Fig. 6). Secondly, the synthesized nanorods and nanopeanuts exhibited anti-biofilm properties, both inhibiting biofilm formation and killing bacteria in already developed biofilms (Figs. 7, 8), which is in agreement with previously published reports^59,60^. Despite these promising data there is however a need for more detailed analyses in which urinary catheters are coated with nanogold; or AuNPs are incorporated into a polymeric catheter material. The potential of the tested nanomaterial as a medical device surface coating indicated by the data is preliminary and certainly has some limitations. However, the shape-controlled activity of gold nanoparticles should be subjected to further analysis for the development of new coating strategies that could considerably contribute to the reduction of hospital-acquired infections.

In summary, the novelty of the presented research lies in a modulation of the synthesis of gold nanoparticles that resulted in the formation of a series of non-spherical gold nanoparticles with substantially reduced bactericidal concentrations, i.e. from µg mL^−1^ to ng mL^−1^, which has not been reported for nanoparticles synthesized by previous methods. The newly synthesized gold nanoparticles therefore provide a unique opportunity to develop more efficient antimicrobial coatings for medical devices and support the treatment of ever-growing antibiotic-resistant urinary tract infections. Notably, the AuP NPs and AuR NPs were effective against all *E. coli* strains, regardless of the identified mechanism of drug resistance. According to previously published data^61^, it is possible that the interaction of nanoparticles with the surface of bacteria is purely physically driven. We suggest that there is an electrostatically-governed association of CTAB-stabilized nanoparticles with the lipopolysaccharides of *E. coli* outer membranes, and this binding initiates the observed killing activity of AuNPs. Assuming that the majority of conventional antibiotics interact with specific targets in the bacterial cell, and bacteria can develop resistance through modification, the non-specific mechanism of the gold nanoparticle action would be favorable for preventing drug resistance.

## Methods

### Materials

Reagents for gold nanoparticle synthesis, including cetrimonium bromide (CTAB) and gold salts were ordered from Sigma-Aldrich (Saint Louis, USA). Colorimetric and fluorescent probes: 3-(4,5-Dimethyl-2-thiazolyl)-2,5-diphenyl-2H-tetrazolium bromide (MTT), resazurin sodium salt, 2ʹ,7ʹ-dichlorofluorescin diacetate (DCFH-DA), *N*-phenyl-1-naphthylamine (NPN), 2-nitrophenyl β-d-galactopyranoside (ONPG), and propidium iodide (PI) were purchased from Sigma-Aldrich (Saint Louis, USA). Conventional antibiotics (amoxicillin/clavuronic acid, ampicillin, cefuroxime, ceftazidime, gentamicin, amikacin, tobramycin, ciprofloxacin, trimethoprim/sulfamethoxazole, nitrofurantoin, and doxycycline) were purchased from OXOID (Hampshire, United Kingdom) and Polfa Tarchomin (Warsaw, Poland).

### Synthesis of spherical, rod-, star-, porous and peanut-shaped gold nanoparticles

The synthesis of rod-shaped gold nanoparticles (AuR NPs) star-shaped gold nanoparticles (AuS NPs), porous-shaped gold nanoparticles (AuSph (70C) NPs) and peanut-shaped gold nanoparticles (AuP NPs) consisted of two steps. First, the solution of gold seeds was prepared as follows: CTAB (0.364 g) was dissolved in H_2_O (5 mL), then HAuCl_4_ (0.85 mg) was dissolved in H_2_O (5 mL) and added to the CTAB solution with vigorous stirring. When the compounds had dissolved, NaBH_4_ (0.6 mL, 0.1 м) was added and the solution became red, which is expected when gold seeds are obtained. In the second step of the synthesis, CTAB (0.364 g) was dissolved in H_2_O (5 mL), then AgNO_3_ (0.2 mL, 3.97 × 10^−3^ м), HAuCl_4_ (5 mL, 5 × 10^−4^ м), C_6_H_8_O_6_ (140 µL, 7.9 × 10^−2^ м) and the gold seed solution obtained in the first step (30 µL) were added to the CTAB solution and mixed with vigorous stirring for 0.5 and 3 h, to obtain AuR NPs and AuP NPs, respectively. The reactions were gone in room temperature. For star AuS NPs the reaction was stopped after 30 min, however, not 70 μL, but 210 μL of 7.8 × 10^−2^ м C_6_H_8_O_6_, was added, while AuSph (70C) NPs was synthesized in the 70 °C. Furthermore, spherical AuNPs was obtained by two different synthesis methods. As one of the spherical Au NPs was used gold seeds obtained in the first step of prepared fancy shape Au NPs (AuSph (CTAB) NPs), while second spherical Au NPs (AuSph NPs) was synthesized as following: HAuCl_4_ with concentration c = 1 mм was used as source of gold ions and trisodium citrate with concentration c = 34 mм (Na_3_C_6_H_5_O_7_) was applied as a reduction and stabilizing agent. Trisodium citrate (980 µL) was heated until boiling on a magnetic stirrer, then the HAuCl_4_ (20 µL) was added. Heating was continued till the color of the solution changed from colorless to pink/red.

### Physicochemical characterization of spherical, rod-, star-, porous and peanut-shaped gold nanoparticles

Scanning transmission electron microscopy (STEM) using a high-angle annular dark-field detector (HAADF) was employed to examine the morphology of the synthesized nanoparticles. Selected area electron diffraction (SAED) patterns were acquired in TEM mode. All measurements were made on an aberration-corrected FEI Titan electron microscope operating at 300 kV, equipped with a FEG cathode. The average size of the longitudinal and transverse axes was calculated from the TEM photos (100 nanoparticles per calculation). Furthermore, optical properties of AuR NPs and AuP NPs was obtained by Ultraviolet–visible spectroscopy (UV–Vis) using Lambda Bio20 instrument from Perkin Elmer. The resolution was chosen to be 1 nm and the scan speed was 240 nm min^−1^. To collect data about the stability of developed nanoparticles, zeta potential values in a pH range from 3.5 to 12.5 were measured using Zetasizer Nano Series from Malvern Instruments. The microelectrophoretic method and Smoluchowski model were employed to determine the zeta potential distribution. Each value was obtained as an average of three subsequent runs of the instrument with at least 20 measurements.

### Antibacterial testing

The bactericidal efficiency of the developed non-spherical nanoparticles was tested using a colony counting assay (killing assay) according to a previously published procedure^37,62^. Briefly, bacterial and fungal cells were grown to mid-log phase at 37 °C, brought to 10^5^ CFU mL^−1^ with sterile phosphate-buffered saline (PBS) and incubated in the presence of developed nanoparticles at concentrations ranging from 0.125 to 1 ng mL^−1^. After 1 h of incubation at 37 °C the plates were transferred to ice and the suspensions were diluted 10- to 1000-fold in PBS. Then, 10 μL aliquots were spotted on Saboraud agar plates (*Candida* isolates), MacConkey agar plates (*E. coli* isolates) or LB agar plates (*P. aeruginosa* and *S. aureus* isolates) for overnight culture at 37 °C and the CFUs were determined. Microbial cell survival after exposure to the tested agent was expressed as log (CFU mL^−1^) or a percentage of the control.

In another set of experiments, the killing efficiency of gold nanoparticles in the presence of highly nutritious growth medium was tested. The minimum inhibitory concentration (MIC), minimum bactericidal concentration (MBC), and minimum biofilm inhibitory concentration (MBIC) were determined. The final concentrations of the tested compounds ranged from 20 µg mL^−1^ to 39 ng mL^−1^ [for AuSph NPs and AuSph (CTAB) NPs] and from 20 to 0.039 ng mL^−1^ [for AuR NPs, AuP NPs, AuS NPs and AuSph (70C) NPs]. The MIC values were determined visually as the lowest concentration of tested agent that showed no microbe growth after 24–48 h of incubation. Fungicidal and bactericidal activity was determined by plating each sample (10 µL) on Saboraud agar plates (*Candida* isolates), MacConkey agar plates (*E. coli* isolates) or LB agar plates (*P. aeruginosa* and *S. aureus* isolates). The minimum biofilm inhibitory concentration was assessed using an MTT test based on the reduction of tetrazolium salts^63^.

### Membrane permeabilization assays

An *N*-phenyl-1-napthylamine (NPN) uptake assay was used to assess the outer layers permeability of fungi and Gram-negative bacteria treated with varied-shaped gold nanoparticles. *C. albicans, E. coli* and *P. aeruginosa* cells were suspended in PBS (OD_600_ ~ 0.5 for fungal and OD_600_ ~ 0.1 for bacterial samples) and then nanoparticles were added at concentrations of 0.5, 5, and 25 µg mL^−1^ (for AuSph NPs) or 0.5, 5, and 25 ng mL^−1^ (for other nanoparticles). NPN was then added to give a final concentration of 0.5 mM, and the mixture was incubated for 5 min. The fluorescence increases due to entering NPN into periplasm space was measured (excitation/emission wavelengths of 348/408 nm) using a Labsystem Varioscan Lux microplate reader (Thermo Fisher Scientific, Waltham, USA). Microscopy visualization of the membrane integrity was carried out using a SYTO9-PI dual staining. Permeabilization of the inner membrane upon treatment with gold nanoparticles was investigated by measuring PI-derived fluorescence signal. The final cell suspension was adjusted to OD_600_ ~ 0.5 or ~ 0.1 prior to addition of gold nanoparticles at doses of 0.5, 5, and 25 µg mL^−1^ (for AuSph NPs) or 0.5, 5, and 25 ng mL^−1^ (for other nanoparticles). The increase of fluorescence signal (excitation/emission wavelengths of 535/617 nm) was determined after 1 h incubation.

### AFM analysis

Atomic force microscopy (AFM) was employed to record the topography of treated bacterial cells for the qualitative assessment of AuNPs-mediated bacterial treatment. Representative strains of *E. coli* and *P. aeruginosa* were resuspended in distilled water (OD_600_ ~ 0.1) and incubated with indicated concentrations of AuR NPs at 37 °C for 1 h. Then, 200 μL bacterial samples were transferred to the mica surface previously functionalized with 0.5% APTES. The attachment of bacterial cells to the mica surface was achieved during 20 min of incubation. Images of bacterial cell surface were collected using a Nano Wizard 4 BioScience AFM (JPK Instruments, Berlin, Germany) operated in Quantitative Imaging Mode. MSNL cantilevers (Bruker, MA, USA) with a nominal spring constant equal to 0.1 N/m were employed. The bacterial cells were located using an optical microscope to collect the topography and adhesion images. Then, 10 μm × 10 μm scanning was performed with the resolution of 128 pixels per line.

### ROS generation assessment

The generation of reactive oxygen species in fungal and bacterial cells upon treatment with spherical and non-spherical gold nanoparticles was determined using 2,7-dichlorofluorescein diacetate (DCFH-DA) as a fluorescent probe^64^. The microbial cultures adjusted to OD_600_ ~ 0.1 or 0.5 were treated with gold nanoparticles at doses of 0.5, 5, and 25 µg mL^−1^ (for AuSph NPs) or 0.5, 5, and 25 ng mL^−1^ (for other nanoparticles) in the presence of DCFH-DA at a final concentration of 20 µM for 1 h at 37 °C. The fluorescence emission of DFCH-DA was measured at 525 nm with an excitation wavelength of 488 nm using a Labsystem Varioscan Lux microplate reader (Thermo Fisher Scientific, Waltham, USA). Untreated fungal and bacterial cells were used as a negative control. Visualization of ROS-positive cells was achieved by DCFH-DA staining and carried out using a fluorescence microscope.

### Investigation of potential of gold nanoparticles as antimicrobial catheters coatings

The ability of rod-shaped gold nanoparticles to limit bacterial attachment and biofilm viability on latex urinary catheters was investigated using a colony counting assay based on a previously published protocol with some modifications^65^. A commercially available latex 20CH Foley urinary catheter (ZARYS International Group Sp. z o.o, Zabrze, Poland) was cut into ~ 4–6 mm fragments under sterile conditions, flushed several times with 96% ethanol, washed thoroughly in PBS, and left under UV light for 30 min. Urine collected from healthy volunteers was filtered through 0.22 µm filters before using in the experiments. Catheter pieces were placed one piece per well in a 24-well plate flooded with bacteria-infected urine (~ 10^7^ CFU mL^−1^ of *E. coli* 369 or *E. coli* 419), and transferred to a rotating incubator for 48 h (37 °C, 70 rpm). To assess the preventive effect of AuP NPs on biofilm formation, nanoparticles with concentrations of 0.5, 5, and 25 ng mL^−1^ were added at day 0. The urine was then replaced once a day without the further addition of nanoparticles. To determine the ability of the nanoparticles to disrupt pre-formed biofilms, nanoparticles were added to wells containing latex urinary catheters on day 2 and left to incubate for a further 24 h. At the final time points, the catheter pieces were transferred to individual test tubes containing 0.5 mL of sterile PBS. The tubes were sonicated for 5 min in a bath sonicator to detach all biofilm-embedded bacteria and thoroughly mixed. After sonication, viable bacteria in PBS from each tube were enumerated after serial dilutions and plated on MacConkey agar plates, as previously described. The values obtained in control cells (without test agents) was taken as 100%.

### Biocompatibility evaluation

Disruption of red blood cells (RBCs) membrane upon treatment with tested nanoparticles was employed as an indicator of toxicity of AuP NPs and AuR NPs against host cells. The hemolytic propertie of the examined nanoparticles was tested using RBCs isolated from the blood of healthy volunteers and suspended in sterile PBS to obtain a hematocrit of ~ 5%. Briefly, peanut- and rod-shaped nanoparticles in doses ranging from 0.125 to 50 ng mL^−1^ were incubated in 96-well plates with the RBC solution at 37 °C for 1 h. After incubation, intact erythrocytes were concentrated by centrifugation (2500 rpm, 10 min) and the supernatant was transferred to a new clear bottom 96-well plate. The release of hemoglobin from damaged RBCs was measured by monitoring the absorbance at 540 nm. Samples exposed to the test agents were compared with wells containing Triton-X100-treated RBCs (denoted 100% hemolysis).

### Investigation of AuP NP and AuR NP killing activity in the presence of urine

In order for the synthesized nanoparticles to be used for the eradication of uropathogens and as coatings for urinary catheters, it is crucial that the appropriate killing efficacy can be achieved in the presence of urine. To assess whether the developed nanomaterials were able to maintain killing effectiveness in the presence of urine, a resazurin-based proliferation assay was performed^66^. Briefly, *E. coli* were suspended in LB broth or LB broth mixed with urine (1:1), brought to OD_600_ ~ 0.1 and incubated in the presence of increasing concentrations of AuP NPs and AuR NPs for 60 min. Owing the high number of bacteria required as a result of the detection limit of the method employed, the final concentrations of AuP NPs and AuR NPs were adjusted to 0.5, 5, and 25 ng mL^−1^. Changes in fluorescence recorded using excitation/emission wavelengths of 520/590 nm were measured using a Labsystem Varioscan Lux microplate reader (Thermo Fisher Scientific, Waltham, USA).

### Influence of pH on the therapeutic efficiency of the developed nanoparticles

To investigate whether differences in the urinary pH influenced nanoparticle efficiency, microdilution assays were performed as described above, but with pH adjustment to pH = 5, 7, or 9 using 0.1 M HCl and 0.1 M NaOH. The pH-dependent changes in antibacterial activity were also compared with those for clinically relevant antibiotics: ciprofloxacin, gentamicin, tobramycin, and doxycycline. The final concentrations of the tested compounds ranged from 10 to 0.019 ng mL^−1^ for AuP NPs and AuR NPs, and from 256 to 0.5 µg mL^−1^ for conventional antibiotics.

### Anti-biofilm properties

The bactericidal efficiency of the peanut- and rod-shaped nanoparticles against *E. coli* cells embedded in biofilms was determined using resazurin-based fluorimetric staining, which allowed the quantification of viable biofilm cells grown in microtiter plates^39^. To evaluate the ability of AuP NPs and AuR NPs to prevent the formation of biofilms, *E. coli* bacteria suspended in LB (OD_600_ ~ 0.1) were added to black bottom 96-well plates and cultivated for 48 h with increasing concentrations of AuP NPs and AuR NPs ranging from 0.125 to 25 ng mL^−1^. To determine the ability of the nanoparticles to disrupt biofilms, agents were added after 48 h of biofilm cultivation and left for incubation for another 1 h. At the end time point, resazurin sodium salt was added to give a final concentration of 0.2 mg mL^−1^. The fluorescence generated after 1 h of incubation at 37 °C was measured at excitation/emission wavelengths of 520/590 nm. The values obtained for control cell cultures (without tested agents) were taken as 100%. The anti-biofilm properties of the developed nanoparticles were compared with the efficiency of ciprofloxacin and gentamicin at concentrations ranging from 0.125 to 25 µg mL^−1^.

### Statistical analysis

The data provided are results from 3 independent experiments ± SD. The significance of differences was determined using the two-tailed Student’s t-test. Statistical analyses were performed using OriginPro 2020 (OriginLab Corporation, Northampton, USA). p < 0.05 was considered to be statistically significant.

### Ethics approval and consent to participate

This study was approved by the Bioethics Committee at the Jan Kochanowski University in Kielce, Faculty of Medicine and Health Sciences (no. 22/2019). Blood and urine samples were collected from adult healthy volunteers under approval of the Bioethics Committee at the Medical University of Bialystok (no. R-I-002/231/2019) and the Jan Kochanowski University in Kielce, Faculty of Medicine and Health Sciences (no. 13/2020), respectively. The study was in accordance with the Declaration of Helsinki and written informed consent was provided by the blood and urine donors.

## Supplementary Information

Supplementary Information.

## Data Availability

All data generated or analyzed during this study are included in this published article (and its Supplementary Information files).

## References

[1] Peach BC, Garvan GJ, Garvan CS, Cimiotti JP (2016). Risk factors for urosepsis in older adults: A systematic review. Gerontol. Geriatr. Med..

[2] Yassin MA, Elkhooly TA, Elsherbiny SM, Reicha FM, Shokeir AA (2019). Facile coating of urinary catheter with bio-inspired antibacterial coating. Heliyon.

[3] Al-Qahtani M, Safan A, Jassim G, Abadla S (2019). Efficacy of anti-microbial catheters in preventing catheter associated urinary tract infections in hospitalized patients: A review on recent updates. J. Infect. Public Health.

[4] Kucharíková S (2016). Covalent immobilization of antimicrobial agents on titanium prevents *Staphylococcus aureus* and *Candida albicans* colonization and biofilm formation. J. Antimicrob. Chemother..

[5] Vester H, Wildemann B, Schmidmaier G, Stöckle U, Lucke M (2010). Gentamycin delivered from a PDLLA coating of metallic implants: In vivo and in vitro characterisation for local prophylaxis of implant-related osteomyelitis. Injury.

[6] McGuffie MJ (2016). Zinc oxide nanoparticle suspensions and layer-by-layer coatings inhibit staphylococcal growth. Nanomedicine.

[7] Jyoti K, Singh A (2017). Evaluation of antibacterial activity from phytosynthesized silver nanoparticles against medical devices infected with. J. Taibah Univ. Med. Sci..

[8] Anyaogu KC, Fedorov AV, Neckers DC (2008). Synthesis, characterization, and antifouling potential of functionalized copper nanoparticles. Langmuir.

[9] Zhang X (2014). Microstructure and cytotoxicity evaluation of duplex-treated silver-containing antibacterial TiO_2_ coatings. Mater. Sci. Eng. C Mater. Biol. Appl..

[10] Polívková M, Hubáček T, Staszek M, Švorčík V, Siegel J (2017). Antimicrobial treatment of polymeric medical devices by silver nanomaterials and related technology. Int. J. Mol. Sci..

[11] Sussman EM, Casey BJ, Dutta D, Dair BJ (2015). Different cytotoxicity responses to antimicrobial nanosilver coatings when comparing extract-based and direct-contact assays. J. Appl. Toxicol..

[12] Pishbin F (2013). Single-step electrochemical deposition of antimicrobial orthopaedic coatings based on a bioactive glass/chitosan/nano-silver composite system. Acta Biomater..

[13] Cioffi N, Torsi L, Ditaranto N, Sabbatini L, Zambonin PG (2004). Antifungal activity of polymer-based copper nanocomposite coatings. Appl. Phys. Lett..

[14] Šlamborová I, Zajícová V, Karpíšková J, Exnar P, Stibor I (2013). New type of protective hybrid and nanocomposite hybrid coatings containing silver and copper with an excellent antibacterial effect especially against MRSA. Mater. Sci. Eng. C Mater. Biol. Appl..

[15] Selvam S (2012). Antibacterial effect of novel synthesized sulfated β-cyclodextrin crosslinked cotton fabric and its improved antibacterial activities with ZnO, TiO_2_ and Ag nanoparticles coating. Int. J. Pharm..

[16] Connor EE, Mwamuka J, Gole A, Murphy CJ, Wyatt MD (2005). Gold nanoparticles are taken up by human cells but do not cause acute cytotoxicity. Small.

[17] Pradeepa (2016). Preparation of gold nanoparticles by novel bacterial exopolysaccharide for antibiotic delivery. Life Sci..

[18] Choi CH, Alabi CA, Webster P, Davis ME (2010). Mechanism of active targeting in solid tumors with transferrin-containing gold nanoparticles. Proc. Natl. Acad. Sci. U. S. A..

[19] Han J (2014). Photothermal therapy of cancer cells using novel hollow gold nanoflowers. Int. J. Nanomed..

[20] Singh M, Harris-Birtill DC, Markar SR, Hanna GB, Elson DS (2015). Application of gold nanoparticles for gastrointestinal cancer theranostics: A systematic review. Nanomedicine.

[21] Chwalibog A (2010). Visualization of interaction between inorganic nanoparticles and bacteria or fungi. Int. J. Nanomed..

[22] Chatterjee T, Chatterjee BK, Chakrabarti P (2017). Modelling of growth kinetics of *Vibrio cholerae* in presence of gold nanoparticles: Effect of size and morphology. Sci. Rep..

[23] Lee H, Lee DG (2018). Gold nanoparticles induce a reactive oxygen species-independent apoptotic pathway in *Escherichia coli*. Colloids Surf. B Biointerfaces.

[24] Ortiz-Benítez EA, Velázquez-Guadarrama N, Durán Figueroa NV, Quezada H, Olivares-Trejo JJ (2019). Antibacterial mechanism of gold nanoparticles on *Streptococcus pneumoniae*. Metallomics.

[25] Penders J, Stolzoff M, Hickey DJ, Andersson M, Webster TJ (2017). Shape-dependent antibacterial effects of non-cytotoxic gold nanoparticles. Int. J. Nanomed..

[26] Hameed S, Wang Y, Zhao L, Xie L, Ying Y (2020). Shape-dependent significant physical mutilation and antibacterial mechanisms of gold nanoparticles against foodborne bacterial pathogens (*Escherichia coli*, *Pseudomonas aeruginosa* and *Staphylococcus aureus*) at lower concentrations. Mater. Sci. Eng. C Mater. Biol. Appl..

[27] Liu M, Guyot-Sionnest P (2005). Mechanism of silver(I)-assisted growth of gold nanorods and bipyramids. J. Phys. Chem. B.

[28] Pedireddy S (2014). One-step synthesis of zero-dimensional hollow nanoporous gold nanoparticles with enhanced methanol electrooxidation performance. Nat. Commun..

[29] Helander IM, Mattila-Sandholm T (2000). Fluorometric assessment of gram-negative bacterial permeabilization. J. Appl. Microbiol..

[30] Kalebina TS, Rekstina VV (2019). Molecular organization of yeast cell envelope. Mol. Biol. (Mosk.).

[31] Piktel E (2020). Rod-shaped gold nanoparticles exert potent candidacidal activity and decrease the adhesion of fungal cells. Nanomedicine (Lond.).

[32] Yang L, Wang K, Li H, Denstedt JD, Cadieux PA (2014). The influence of urinary pH on antibiotic efficacy against bacterial uropathogens. Urology.

[33] Lai HC (2019). Association between urine pH and common uropathogens in children with urinary tract infections. J. Microbiol. Immunol. Infect..

[34] Mobley HL, Hausinger RP (1989). Microbial ureases: Significance, regulation, and molecular characterization. Microbiol. Rev..

[35] Wnorowska U (2015). Extracellular DNA as an essential component and therapeutic target of microbial biofilm. Med. Stud. Studia Medyczne.

[36] Costerton JW, Stewart PS, Greenberg EP (1999). Bacterial biofilms: A common cause of persistent infections. Science.

[37] Wnorowska U (2019). Use of ceragenins as a potential treatment for urinary tract infections. BMC Infect. Dis..

[38] Sarker SD, Nahar L, Kumarasamy Y (2007). Microtitre plate-based antibacterial assay incorporating resazurin as an indicator of cell growth, and its application in the in vitro antibacterial screening of phytochemicals. Methods.

[39] Van den Driessche F, Rigole P, Brackman G, Coenye T (2014). Optimization of resazurin-based viability staining for quantification of microbial biofilms. J. Microbiol. Methods.

[40] Pal S, Tak YK, Song JM (2007). Does the antibacterial activity of silver nanoparticles depend on the shape of the nanoparticle? A study of the Gram-negative bacterium *Escherichia coli*. Appl. Environ. Microbiol..

[41] Acharya D (2018). Shape dependent physical mutilation and lethal effects of silver nanoparticles on bacteria. Sci. Rep..

[42] Slomberg DL (2013). Role of size and shape on biofilm eradication for nitric oxide-releasing silica nanoparticles. ACS Appl. Mater. Interfaces.

[43] Shamaila S (2016). Gold nanoparticles: An efficient antimicrobial agent against enteric bacterial human pathogen. Nanomaterials (Basel)..

[44] Kohout C, Santi C, Polito L (2018). Anisotropic gold nanoparticles in biomedical applications. Int. J. Mol. Sci..

[45] Mahmoud NN, Alkilany AM, Khalil EA, Al-Bakri AG (2017). Antibacterial activity of gold nanorods against. Int. J. Nanomed..

[46] Nakata K, Tsuchido T, Matsumura Y (2011). Antimicrobial cationic surfactant, cetyltrimethylammonium bromide, induces superoxide stress in *Escherichia coli* cells. J. Appl. Microbiol..

[47] Castillo-Martínez J (2015). Antibacterial and antibiofilm activities of the photothermal therapy using gold nanorods against seven different bacterial strains. J. Nanomater..

[48] Akhavan O, Ghaderi E (2010). Toxicity of graphene and graphene oxide nanowalls against bacteria. ACS Nano.

[49] Maleki Dizaj S, Mennati A, Jafari S, Khezri K, Adibkia K (2015). Antimicrobial activity of carbon-based nanoparticles. Adv. Pharm. Bull..

[50] Padmavathy N, Vijayaraghavan R (2008). Enhanced bioactivity of ZnO nanoparticles-an antimicrobial study. Sci. Technol. Adv. Mater..

[51] Cui Y (2012). The molecular mechanism of action of bactericidal gold nanoparticles on *Escherichia coli*. Biomaterials.

[52] Lee B, Lee DG (2019). Synergistic antibacterial activity of gold nanoparticles caused by apoptosis-like death. J. Appl. Microbiol..

[53] Zheng Y (2018). Mercaptopyrimidine-conjugated gold nanoclusters as nanoantibiotics for combating multidrug-resistant superbugs. Bioconjug. Chem..

[54] Xie Y (2018). Gold nanoclusters for targeting methicillin-resistant *Staphylococcus aureus* in vivo. Angew. Chem. Int. Ed. Engl..

[55] Mohamed MM, Fouad SA, Elshoky HA, Mohammed GM, Salaheldin TA (2017). Antibacterial effect of gold nanoparticles against. Int. J. Vet. Sci. Med..

[56] Nasser F, Davis A, Valsami-Jones E, Lynch I (2016). Shape and charge of gold nanomaterials influence survivorship, oxidative stress and moulting of *Daphnia magna*. Nanomaterials (Basel)..

[57] Singha P, Locklin J, Handa H (2017). A review of the recent advances in antimicrobial coatings for urinary catheters. Acta Biomater..

[58] Vranic SM, Zatric N, Rebic V, Aljicevic M, Abdulzaimovic A (2017). The most frequent isolates from outpatients with urinary tract infection. Mater. Sociomed..

[59] Yu Q (2016). Inhibition of gold nanoparticles (AuNPs) on pathogenic biofilm formation and invasion to host cells. Sci. Rep..

[60] Ramasamy M, Lee JH, Lee J (2017). Development of gold nanoparticles coated with silica containing the antibiofilm drug cinnamaldehyde and their effects on pathogenic bacteria. Int. J. Nanomed..

[61] Jacobson KH (2015). Lipopolysaccharide density and structure govern the extent and distance of nanoparticle interaction with actual and model bacterial outer membranes. Environ. Sci. Technol..

[62] Durnaś B (2016). Candidacidal activity of selected ceragenins and human cathelicidin LL-37 in experimental settings mimicking infection sites. PLoS One.

[63] Niemirowicz K (2017). Formulation and candidacidal activity of magnetic nanoparticles coated with cathelicidin LL-37 and ceragenin CSA-13. Sci. Rep..

[64] Ong KS, Cheow YL, Lee SM (2017). The role of reactive oxygen species in the antimicrobial activity of pyochelin. J. Adv. Res..

[65] Narayanan A, Nair MS, Muyyarikkandy MS, Amalaradjou MA (2018). Inhibition and inactivation of uropathogenic. Int. J. Mol. Sci..

[66] Bucki R (2019). Susceptibility of microbial cells to the modified PIP. J. Nanobiotechnol..

