# Supplementary material for: Varied-shaped gold nanoparticles with nanogram killing efficiency as potential antimicrobial surface coatings for the medical devices
Source: Sci Rep. 2021 Jun 15;11:12546. doi: 10.1038/s41598-021-91847-3 (PMC8206335; doi:10.1038/s41598-021-91847-3)
Supplement: Supplementary file 1 — Supplementary Information. [file 41598_2021_91847_MOESM1_ESM.pdf]

Varied-shaped gold nanoparticles with nanogram killing efficiency as potential antimicrobial surface coatings for the medical devices

Ewelina Piktel, Łukasz Suprewicz, Joanna Depciuch, Sylwia Chmielewska, Karol Skłodowski, Tamara Daniluk, Grzegorz Król, Paulina Kołat-Brodecka, Piotr Bijak, Anna Pajor-Świerzy, Krzysztof Fiedoruk, Magdalena Parlinska-Wojtan and Robert Bucki

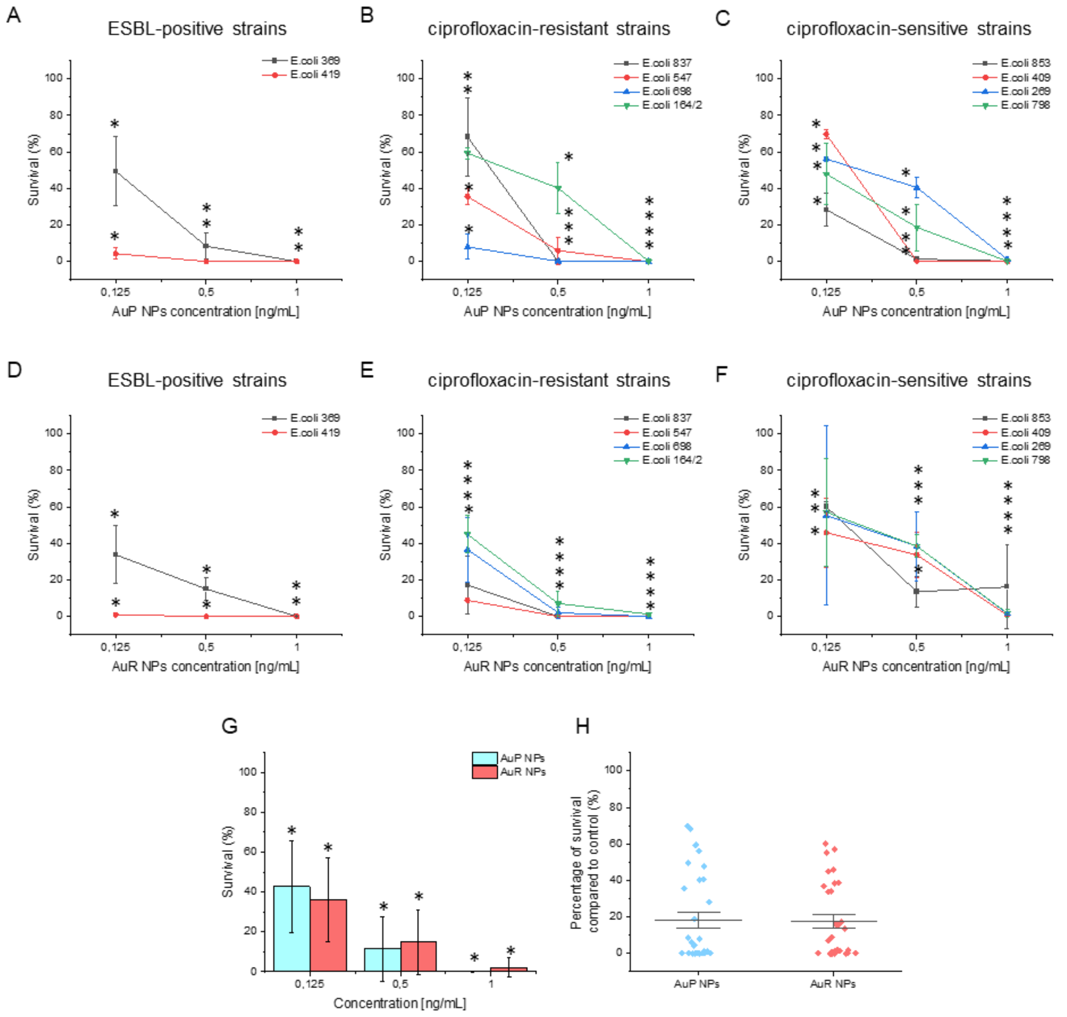

Supplementary Figure 1. Bactericidal activity of peanut- (AuP NPs; panels A–C) and rod-shaped (AuR NPs; panels D–F) gold nanoparticles against clinical isolates of *Escherichia coli* obtained from patients diagnosed with urinary tract infections. The killing activity of the non-spherical nanoparticles at concentrations of 0.125, 0.5, and 1 ng mL^{-1} was tested using a colony counting assay against ESBL-positive strains (panel A and D; *E.coli* 369 and 419), ciprofloxacin-resistant strains (panel B and E; *E.coli* 837, 547, 698, and 164/2) and ciprofloxacin-sensitive strains (panel C and F; *E.coli* 853, 409, 269, and 798). Panel G shows the killing assay results for 10 UTI-associated *E.coli* strains as average values. The statistical analysis of the differences in killing abilities of AuP NPs and AuR NPs is presented in panel H. Results are presented as mean ± SD for 3–20 measurements. * indicates statistical significance (p<0.05) when compared to untreated bacteria.

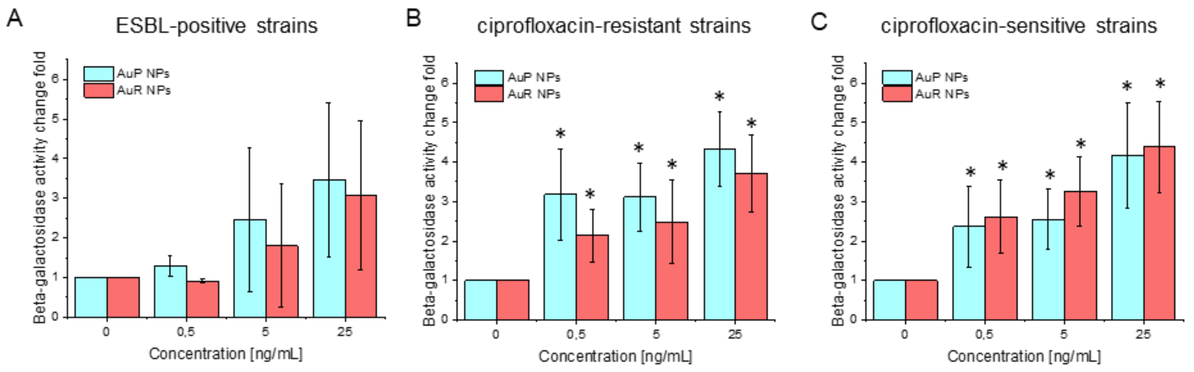

Supplementary Figure 2. Release of β-galactosidase from ESBL-positive (panel A), ciprofloxacin-resistant (panel B) and ciprofloxacin-sensitive *E.coli* bacteria (panel C) upon treatment with non-spherical gold nanoparticles at concentrations of 0.5, 5 and 25 ng mL^{-1}. Results are presented as mean ± SD for 3 measurements. * indicates statistical significance (p<0.05) when compared to untreated bacteria.

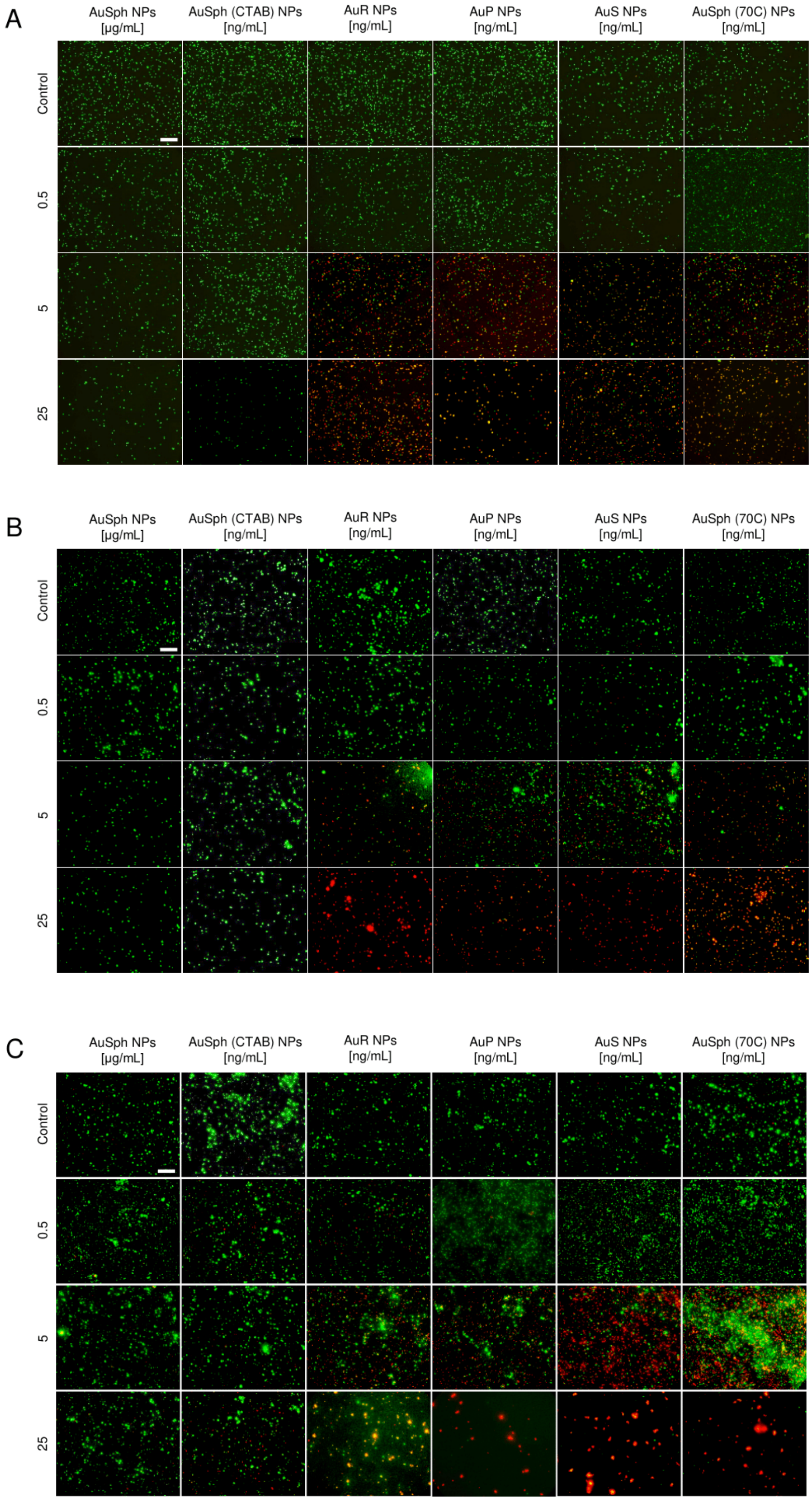

Supplementary Figure 3. Decrease of viability of *C. albicans* (panel A), *E. coli* (panel B) and *P. aeruginosa* (panel C) followed incubation with developed nanoparticles evaluated using fluorescence microscopy. Scale bar ~ 50 µm (panel A) and 200 µm (panels B and C).

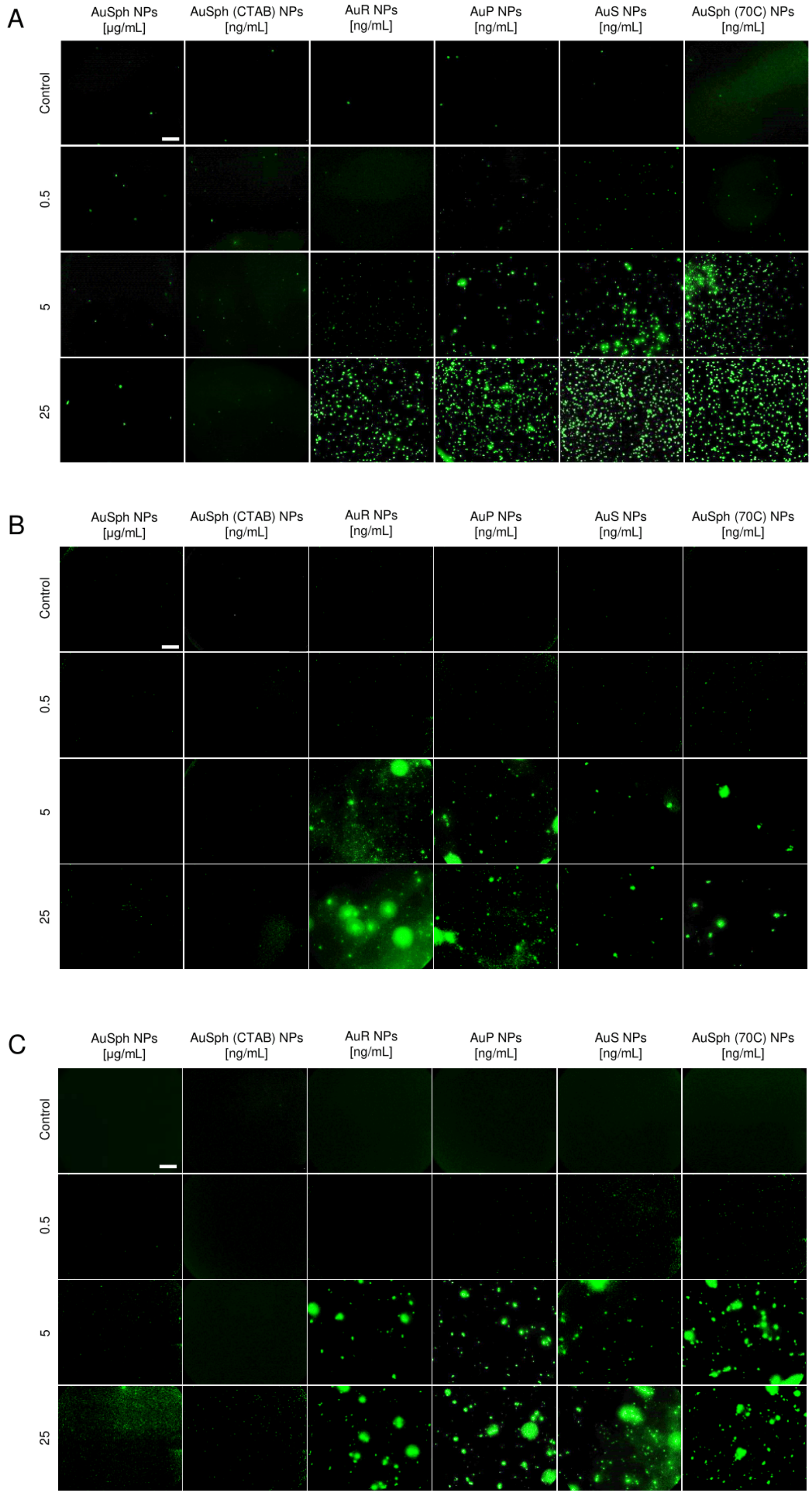

Supplementary Figure 4. Increase of ROS-positive cells of *C. albicans* (panel A), *P. aeruginosa* (panel B) and *S. aureus* (panel C) followed incubation with developed nanoparticles evaluated using fluorescence microscopy. Scale bar ~ 50 µm (panel A) and 200 µm (panels B and C).

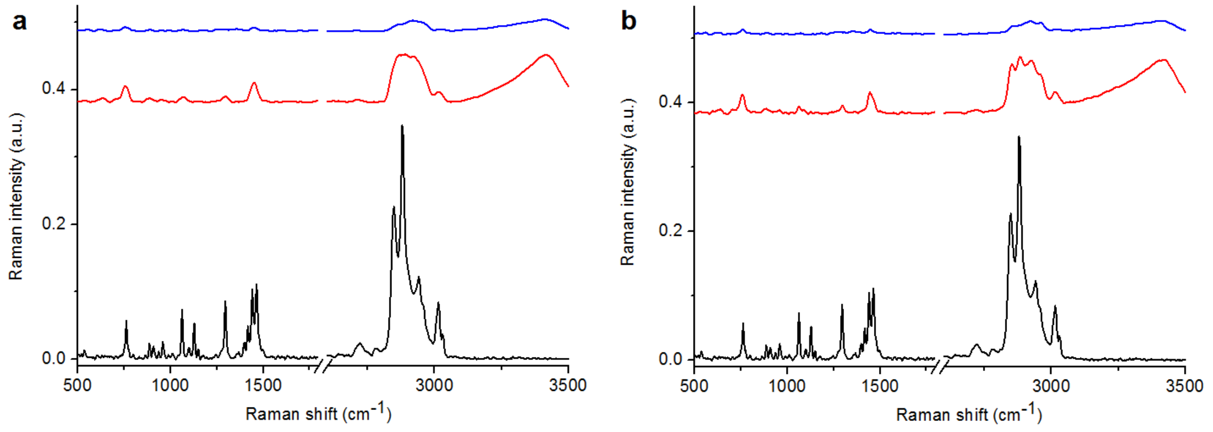

Supplementary Figure 5. FT-Raman spectra of pure CTAB (black spectrum); nanoparticles before (red spectrum); and after (blue spectrum) rinsing the CTAB from the solution of AuP NPs (panel A) and AuR NPs (panel B).

Supplementary Table 1. Clinical characteristic of *Escherichia coli* strains used in the study.

NO.	AGE	SEX	BACTERIURIA
**164/2**	4	F	10^7 CFU/mL
**853**	1.5	F	10^4 CFU/mL
**798**	7 mos.	M	10^5 CFU/mL
**698**	58	M	$>10^7$ CFU/mL
**547**	19	F	$>10^7$ CFU/mL
**419**	29	F	$>10^7$ CFU/mL
**409**	1	F	10^7 CFU/mL
**369**	84	F	$>10^7$ CFU/mL
**269**	6 mos.	M	10^7 CFU/mL
**837**	3	F	10^4 CFU/mL

Supplementary Table 2. Antibiotic sensitivity of clinical strains of *Escherichia coli* used in this study. The antimicrobial inhibition zones were measured and recorded according to EUCAST and CLSI (for doxycycline) standards. R - resistant; S- sensitive; I-intermediate

	AMC	AM	CXM	CAZ	GM	AN	TB	CIP	SXT	F/M	DOX
**164/2**	S	R	S	S	S	S	R	R	R	S	R
**853**	R	R	S	S	S	S	R	S	R	S	I
**798**	S	R	S	S	I	S	R	S	I	R	R
**698**	R	R	S	S	S	S	S	R	S	S	R
**547**	R	R	S	S	S	S	R	R	R	S	R
**419**	S	R	R	R	S	S	R	R	R	S	I
**409**	S	R	S	S	S	S	I	S	R	S	I
**369**	S	R	R	R	S	S	R	R	R	S	R
**269**	S	R	S	S	S	S	I	S	S	S	S
**837**	R	R	S	S	S	S	I	R	R	S	R

Abbreviations: AMC: amoxicillin/clavulanic acid; AM: ampicillin; CXM: cefuroxime; CAZ: ceftazidime; GM: gentamycin; AN: amikacin; TB: tobramycin; CIP: ciprofloxacin; SXT: trimethoprim/sulfamethoxazole; F/M: nitrofurantoin; DOX: doxycyclinum

Supplementary Table 3. Minimal inhibitory concentrations (MIC; ng mL^{-1} or µg mL^{-1}), minimal bactericidal concentrations (MBC; ng mL^{-1} or µg mL^{-1}) and minimal biofilm inhibitory concentrations (MBIC; ng mL^{-1} or µg mL^{-1}) of peanut- or rod-shaped nanoparticles (AuP NPs, AuR NPs, respectively) against 10 clinical isolates of *Escherichia coli* recorded in a medium with pH 5, 7 or 9.

		MIC/MBC/MBIC [NG ML^{-1}]	
		**AUP NPS**	**AUR NPS**
***E. coli* 164/2**	pH=5	0.31 / 0.62 / 0.62	0.31 / 0.62 / 0.62
	pH=7	0.31 / 0.31 / 0.31	0.31 / 0.31 / 0.31
	pH=9	0.16 / 0.16 / 0.16	0.16 / 0.16 / 0.16
***E. coli* 853**	pH=5	0.31 / 0.62 / 0.62	0.31 / 0.62 / 0.62
	pH=7	0.31 / 0.31 / 0.62	0.31 / 0.31 / 0.31
	pH=9	0.16 / 0.16 / 0.31	0.16 / 0.16 / 0.16
***E. coli* 798**	pH=5	0.31 / 0.62 / 0.62	0.31 / 0.31 / 0.62
	pH=7	0.31 / 0.31 / 0.62	0.31 / 0.31 / 0.31
	pH=9	0.16 / 0.16 / 0.16	0.16 / 0.16 / 0.16
***E. coli* 698**	pH=5	0.31 / 0.62 / 0.62	0.31 / 0.62 / 0.62
	pH=7	0.31 / 0.31 / 0.62	0.31 / 0.31 / 0.62
	pH=9	0.16 / 0.16 / 0.16	0.08 / 0.16 / 0.16
***E. coli* 547**	pH=5	0.62 / 0.62 / 0.62	0.62 / 0.62 / 0.62
	pH=7	0.62 / 0.62 / 0.62	0.62 / 0.62 / 1.25
	pH=9	0.16 / 0.31 / 0.31	0.16 / 0.31 / 0.62
***E. coli* 419**	pH=5	0.62 / 0.62 / 1.25	0.62 / 0.62 / 0.62
	pH=7	0.31 / 0.62 / 0.62	0.31 / 0.31 / 0.31
	pH=9	0.16 / 0.16 / 0.16	0.16 / 0.16 / 0.62
***E. coli* 409**	pH=5	0.62 / 0.62 / 0.62	0.62 / 0.62 / 1.25
	pH=7	0.31 / 0.31 / 0.62	0.31 / 0.31 / 0.62
	pH=9	0.16 / 0.16 / 0.31	0.16 / 0.16 / 0.16
***E. coli* 369**	pH=5	0.31 / 0.62 / 0.62	0.31 / 0.31 / 0.62
	pH=7	0.62 / 0.62 / 1.25	0.62 / 0.62 / 0.62
	pH=9	0.16 / 0.31 / 0.31	0.08 / 0.16 / 0.16
***E. coli* 269**	pH=5	0.62 / 0.62 / 1,25	0.31 / 0.62 / 0.62
	pH=7	0.62 / 0.62 / 0.62	0.62 / 0.62 / 1.25
	pH=9	0.16 / 0.16 / 0.31	0.16 / 0.16 / 0.62
***E. coli* 837**	pH=5	0.31 / 0.62 / 0.62	0.31 / 0.62 / 1.25
	pH=7	0.31 / 0.31 / 0.31	0.31 / 0.62 / 0.62
	pH=9	0.08 / 0.08 / 0.08	0.08 / 0.16 / 0.31
